# The YOUth study: Rationale, design, and study procedures

**DOI:** 10.1016/j.dcn.2020.100868

**Published:** 2020-10-07

**Authors:** N. Charlotte Onland-Moret, Jacobine E. Buizer-Voskamp, Maria E.W.A. Albers, Rachel M. Brouwer, Elizabeth E.L. Buimer, Roy S. Hessels, Roel de Heus, Jorg Huijding, Caroline M.M. Junge, René C.W. Mandl, Pascal Pas, Matthijs Vink, Juliëtte J.M. van der Wal, Hilleke E. Hulshoff Pol, Chantal Kemner

**Affiliations:** a Julius Center for Health Sciences and Primary Care, University Medical Center Utrecht, Utrecht University, Utrecht, the Netherlands; b Faculty of Social and Behavioural Sciences, Utrecht University, Utrecht, the Netherlands; c Division of Woman and Baby, University Medical Center Utrecht, Utrecht University, the Netherlands; d Division of Biomedical Genetics, University Medical Center Utrecht, Utrecht University, the Netherlands; e UMC Utrecht Brain Center, University Medical Centre Utrecht, Utrecht University, Utrecht, the Netherlands; f Experimental Psychology, Helmholtz Institute, Utrecht University, Utrecht, the Netherlands; g Developmental Psychology, Utrecht University, Utrecht, the Netherlands; h Dept. Clinical Child and Family Studies, Social and Behavioral Sciences, Utrecht Univerity, Utrecht, the Netherlands

**Keywords:** Mental health, Cognitive development, Social competence, Self-regulation, Longitudinal cohorts study, Birth cohort

## Abstract

Behavioral development in children shows large inter-individual variation, and is driven by the interplay between biological, psychological, and environmental processes. However, there is still little insight into how these processes interact. The YOUth cohort specifically focuses on two core characteristics of behavioral development: social competence and self-regulation. Social competence refers to the ability to engage in meaningful interactions with others, whereas self-regulation is the ability to control one’s emotions, behavior, and impulses, to balance between reactivity and control of the reaction, and to adjust to the prevailing environment. YOUth is an accelerated population-based longitudinal cohort study with repeated measurements, centering on two groups: YOUth Baby & Child and YOUth Child & Adolescent. YOUth Baby & Child aims to include 3,000 pregnant women, their partners and children, wheras YOUth Child & Adolescent aims to include 2,000 children aged between 8 and 10 years old and their parents. All participants will be followed for at least 6 years, and potentially longer.

In this paper we describe in detail the design of this study, the population included, the determinants, intermediate neurocognitive measures and outcomes included in the study. Furthermore, we describe in detail the procedures of inclusion, informed consent, and study participation.

## Highlights

• This article describes the rationale, design, and procedures of the YOUth cohort.
• YOUth is set up to investigate what drives the development of social competence and self-regulation in children.
• YOUth specifically investigates the role of neurocognitive development in child development.
• YOUth has a flexible longitudinal design with repeated measurements throughout childhood, starting prenatally.

## 1 Rationale for YOUth

The YOUth cohort specifically focuses on two core characteristics of behavioral development: social competence and self-regulation. Social competence refers to the ability to engage in meaningful interactions with others, whereas self-regulation is the ability to control one’s emotions, behavior, and impulses, to balance between reactivity and control of the reaction, and to adjust to the prevailing environment. The importance of these two components in behavioral development and their relevance for this study have been described in detail in two other papers in this issue [In this special issue: Junge et al., 2020; Vink et al., 2020].

In brief, the development of self-regulation and social competence in children shows large inter-individual variation (Nesselroade and Molenaar, 2010). We know that the development of these components of behavioral development in children is driven by the interplay between biological, psychological, and environmental processes. However, there is still little insight into how these processes interact. Therefore, up to now, it has been virtually impossible to predict which combination of factors explains individual variability in the development of self-regulation and social competence.

In-depth understanding of why there are major individual differences in behavioral development, and more specifically the development of self-regulation and social competence, is hampered greatly by the traditional boundaries of the scholarly disciplines involved. On the one hand, there are longitudinal studies that investigate the effects of psychological child characteristics and environmental factors on development [e.g. van Eijsden et al., 2011; Heude et al., 2016; Connelly and Platt, 2014; Ormel et al., 2012]. However, these studies typically lack a deeper understanding of the biological and brain mechanisms through which such factors affect behavioral development. On the other hand, despite the obvious relevance of brain development for self-regulation and social competence, there is a paucity of longitudinal studies examining neurocognitive development together with structure and function of the brain in childhood. Most evidence originates from tightly controlled cross-sectional studies in small sample sizes (Greven et al., 2015; Wyciszkiewicz et al., 2017; Ambrosino et al., 2017; Noordermeer et al., 2016; Rogers and De Brito, 2016; Stanfield et al., 2008; Tamura et al., 2010). As a result, there is little insight into how biological, child-related and environmental factors interact in shaping brain and behavior during the course of development. To promote child (and future adult) mental health, we need more knowledge on the role of individual attributes (e.g. genetic and biological factors), social and economic circumstances, and environmental factors in neurocognitive, and subsequent behavioral development, and on how these factors interact.

**Fig. 1:**
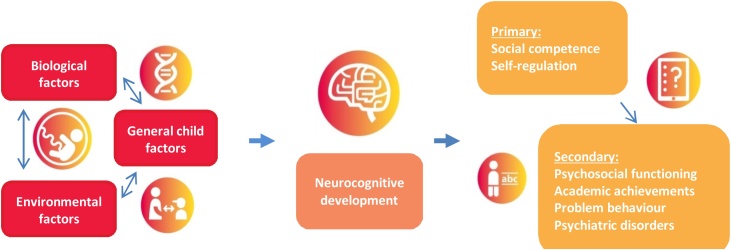
YOUth research question.

To address this gap in our knowledge, the YOUth cohort aims to investigate how neurocognitive development in the general population mediates the influence of biological, child-related and environmental determinants on the development of self-regulation and social competence by following children from pregnancy until their early adulthood (Fig. 1). By extensively mapping the general variation in typical development from different perspectives (determinants, mediating neurocognitive mechanisms, specific behavioral outcomes in social competence and self-regulation, and general functioning), the overall objective of this cohort study is to understand the role of neurocognitive development in the development of social competence and self-regulation. Furthermore, YOUth aims to identify children at high risk of having developmental problems later in life, which can be very broad ranging from learning disabilities to more psychiatric disorders. Given the variety in collected data, YOUth is also very well suited to develop prediction models that predict behavior from environmental- and biological determinants and neurocognitive developmental features.

## 2 Study design

YOUth is a population-based prospective longitudinal cohort study with repeated measures in Utrecht and its surrounding areas. YOUth has an accelerated longitudinal design, including broad age ranges. Inclusion starts at two critical moments in a child’s life: during pregnancy (YOUth Baby & Child) and just before puberty (YOUth Child & Adolescent). The main advantage of an accelerated longitudinal design is that it enables us to span the complete age range in a shorter amount of time (Galbraith et al., 2017). Moreover, as this design leads to a shorter study design, it has been suggested that the amount of drop-out will be reduced (Galbraith et al., 2017). A potential disadvantage is that cohort effects can still be present and this may lead to potential problem with respect to missing data introduced by the design. However, it has been shown that 30 % loss-to-follow up resulted in only 7% power loss, and that with this design cohort effects can be estimated and accounted for (Galbraith et al., 2017). Another potential disadvantage is that, because not all children have measurements at all ages, some of the comparisons are not truly within participant comparisons

YOUth also uses a flexible design. This means that we will have repeated measurements for all children with a fixed time interval (3 years), but not all children will be measured at the same age (3-years age ranges). The main benefit of this flexible design is that it provides more detailed information that covers the range of typical development over time.

Children’s brains and neurocognitive functions develop fast prenatally and in the first years after birth, but continue into adolescence (Johnson, 2000; Fox et al., 2010). Thus, having only a single measurement in the first few years of life may not be enough to obtain valid estimates of the developmental growth curves in this period. Therefore, we have decided to measure children more frequently in the first two years after conception, and in smaller age ranges (i.e. 20–24 weeks and 29–33 weeks gestational weeks, and 4–6 months old and 9–11 months old). After the age of two, the speed of development decreases, which allows for more time between measurements. Hence, after the age of two the frequency of the measurements decreases and the age ranges are broadened to the standard 3-years age interval that is used for the rest of the follow-up period (i.e. 2–4 years old, 5–7 years old, etc.). The age at which the children are asked to return for the measurement wave at 2–4 years old is assigined randomly, in order to end up with an approximately flat distribution of ages included in this wave. After this wave, children return after fixed 3-years intervals.

**Fig. 2:**
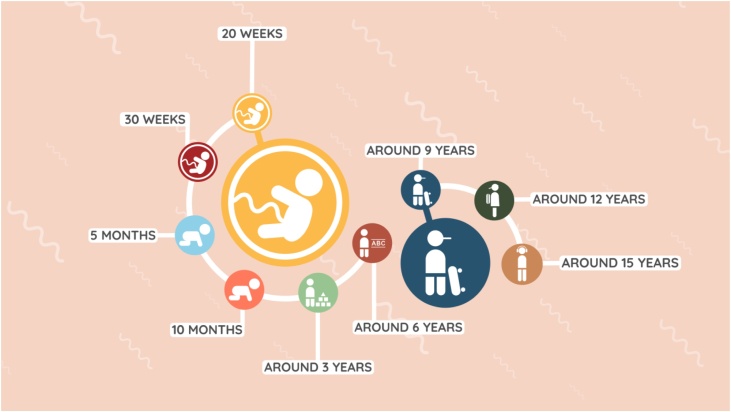
YOUth study design. ‘Around pregnancy’: 20–24 weeks of pregnancy and 29–33 weeks of pregnancy. Around 0’: 4–6 months and 9–11 months. ‘Around 3’: 2–4 years. ‘Around 6’: 5–7 years. ‘Around 9’: 8–10 years. ‘Around 12’: 11–13 years. ‘Around 15’: 14–16 years.

Fig. 2 shows the measurement waves in YOUth. We have in total 9 visits in 7 measurement waves in both cohorts together; 6 visits in YOUth Baby & Child and 3 visits in YOUth Child & Adolescent. Each measurement wave is named differently (‘Around pregnancy’,’ Around 0’, ‘Around 3’, etc.) ‘Around pregnancy’ and ‘Around 0’ both include 2 visits to the center at 20- and 30 weeks of pregnancy for ‘Around pregnancy’, and at 4–6 and 9–11 months old for ‘Around 0’. All other measurement waves include one visit to our Child Research Center. At birth, there is no formal visit, but we ask the midwives to collect cord blood and the mothers to fill in a short questionnaire. For now, the visits for YOUth Baby & Child (stops at ‘Around 6’) do not yet overlap with those of YOUth Child & Adolescent (starts at ‘Around 9’), as additional funding needs to be recuired first. However, it is explicitly the purpose of doing this to be able to disentangle cohort effects from real developmental effects.

## 3 Study population

### 3.1 Setting

YOUth stands for “Youth Of Utrecht” as we aim to include a population-based sample of 3,000 pregnant women and approximately 2,000 children aged 8, 9 and 10 years old from Utrecht and its surrounding areas. The region from which the participants are recruited is a densely populated region that combines both urban and rural areas, and covers the province of Utrecht and a few cities on the borders of this province. Overall, the province of Utrecht consists of 1,3 Million inhabitants, with its major, and growing, city (also called) Utrecht consisting currently of 357.000 inhabitants. Thus, the catchment area of YOUth represents approximately 7.6 % of the Dutch population of currently over 17 Million inhabitants. Approximately 16 % of the inhabitants of the province of Utrecht are 0–10 years old and on average 14,500 babies are born each year. Compared to the rest of the Netherlands, inhabitants of the province of Utrecht are relatively highly educated; In 2015 approximately 38 % of the population was highly educated compared to 28 % in the rest of the country.

All measurements are conducted in dedicated labs in the Child Research Center at the Utrecht Science Park.

YOUth is approved by the Medical Research Ethics Committee Utrecht.

### 3.2 In- and exclusion criteria

As YOUth aims to investigate the range of typical behavioral development, all pregnant women and children in the described age ranges can be included. YOUth includes children and their parents. Parents are considered those with parental authority over the child. Children are only excluded if they are not mentally or physically capable of performing the tests during the visits to our center. Moreover, all participants, including the parents in both subcohorts, should master the Dutch language sufficiently to be able to understand all information and instructions.

We opted to include only one child per family. Inclusion of siblings as part of the design has some advantages, but several disadvantages as well. Advantages include for instance, the possibility to estimate within and between family effects, and the possibility to estimate the contribution of the non-shared environment. However, potential disadvantages are, for instance, that the power to estimate direct associations is lower, introducing confounding and selection biases. Weighing these advantages and disadvantages, for logistic reasons, and the fact that within family effects are not the main goal of YOUth, we decided against including siblings in our study. As a result, in YOUth Baby & Child, in case of a twin pregnancy only one child per twin pair participates from ‘Around 0′ onwards. In both cohorts the choice of which child will participate is made by the parents.

Pregnant women and children are also excluded if the parents do not allow us to report unexpected findings back to them or to their general practitioners. Unexpected findings are those findings that are the result of the scientific research performed in YOUth, which are relevant for the future health of the participant or its family. We expect these findings to be rare, as YOUth is not set-up as a medical screening, and as such there is no active search for these findings.

In YOUth Child & Adolescent, if children have interfering metal objects in or around the body (e.g. braces) they can be included to participate in most aspects of the study. Only the magnetic resonance imaging (MRI) measurements are omitted in that case.

### 3.3 Informed consent and testing day procedures

Before the first measurement wave parents receive the information package. This package contains an extensive information letter explaining the study, two informed consent forms for both parents for their own data, and one informed consent form for the data of the child to be signed by the legal representatives of that child, and a reply envelope. Informed consent is obtained from both parents at each measurement wave. For the children in YOUth Child & Adolescent a social story (a visual story describing what the child can expect on a testing day and explaining the study in a way they can understand) is added to the information package. At subsequent measurement waves, only a brief description of the measurement day and the informed consent forms for that measurement wave are sent to the parents. From age 12 to 15, the children are asked for written consent as well, together with the legal representatives. From age 16 and older only the child is asked for written consent. Furthermore, we work according to the code of conduct for minors, as was drawn up by the Netherlands Association for Paediatric Medicine (NVK, 21 May 2001). This means that we make sure that, before we start the measurements, the children are willing to participate. The study procedures are ended if the participant shows signs of resistance. Participants can terminate their participation at any time for any reason without any consequences. The investigator can also decide to withdraw a subject at any moment because he/she does not meet the inclusion criteria. As it is difficult for a baby or toddler to indicate their refusal to participate in a study, we continuously monitor the child during the testing. If, according to the opinion of the parent or the researcher, the behavior of the child differs negatively (crying, fussiness, restlessness) from what is normally observed in the child, the assessments will be aborted. At all times during assessments with young children, the parent stays with the child.

Prior to the measurements a testing day begins with a research assistant explaining the study procedures and obtaining informed consent. Participants are encouraged to ask questions. The research assistant then checks whether all legal representatives have signed the informed consent forms, and subsequently signs the forms in the presence of the participant. Not all legal representatives have to be present at the testing day, but in the case that only one accompanies the child, which is usually the case, the other representative must have signed the consent form of the child at home. In the case that only one person has the legal authority over the child, only this person has to sign the consent form of the child.

All children visit the Child Research Center several times for extensive measurements. The sequence in which the measurements are taken differs for the children. Children are more tired at the end of the day, while at the same time the effect of tiredness within a child diminishes in later rounds when the children get older. Having the measurements in a standard order would therefore lead to a systematic bias towards tiredness in a specific experiment, due to the timing at which the measurement is taken. Having different sequences enables us to adjust for the confounding effect of tiredness and changes therein over time. Children are allocated to different sequences as randomly as possible. At regular intervals the logistics team checks whether all different sequences are uniformly used.

At each measurement wave, both parents receive several online questionnaires about themselves, and one parent is also asked to fill in questionnaires about the child. Some of the questionnaires are filled in during the testing days while others are sent later to reduce the burden for the parents and the child. As the children grow older, the amount of questionnaires for the child increases and the amount of questionniares for the parents decreases. To lower the burden for the children, from ‘Around 12’ on we do not administer all questionnaires at the same time. The total amount of questionnaires is split in three equal parts (A, B, and C). Each part is sent to one third of the participating children at the measurement day such that all questionnaires are filled in by at least one part of the children around the measurement day. Then one year later, all participating children receive another part that they had not yet filled in and again one year later they receive the last part. Each set contains the social competence and self-regulation measures (see Section 5.5) and the puberty development scale. Hence, these four questionnaires are measured yearly. This protocol for administering questionnaires has certain advantages, as this reduces the burden of questionnaires for the children, while at the same time all questionnaires are administered in part at the same time as the measurement day. This enables us to study the associations of each questionnaire with all measurements of the testing day in at least a part of our population. Moreover, we have shorter intervals between the measurements for all questionnaire data (Graham et al., 2006).

## 4 Recruitment and follow-up procedures

### 4.1 YOUth baby & child

For YOUth Baby & Child we aim to include 3,000 pregnant women, the partners of these women and 3,000 babies born from these pregnancies. Inclusion starts prenatally. In the Netherlands, the primary care for low risk pregnant women is generally performed by midwifes. A pregnant woman chooses her own midwife who provides pre-, peri- and postnatal care. In total, 32 midwifery practices are participating in YOUth. In addition, the primary care midwifery practices of five large hospitals in the region participate in YOUth as well.

Eligible pregnant women for YOUth are recruited through these midwifery practices. Midwives hand flyers to the pregnant women at their first visit, and provide some background information when time allows. If a woman is interested to participate she sends in the reply card, goes to the YOUth website (www.youthonderzoek.nl), or replies by telephone or e-mail. After doing so, she receives an extensive information brochure explaining the study, the informed consent forms, and a reply envelope by mail. Within two weeks the woman is contacted by phone to answer any questions she or (if applicable) her partner has. When, after carefully reading the materials, the woman and partner decide to participate, in- and exclusion criteria are checked through telephone screening and the first appointment is scheduled.

We have no information on the number of flyers that are handed out. However, of those women requesting an information brochure, 60–65 % are currently being included in the study.

#### 4.1.1 ‘Around pregnancy’: baseline and 1st follow-up visit of YOUth Baby & Child

Our baseline visit and first follow-up visit take place in our first measurement wave ‘Around pregnancy’. The first appointment between 20 and 24 weeks of gestational age is scheduled after the woman has had her regular 20-weeks anatomical medical ultrasound, in order to reduce the chance of finding serious congenital malformations during their visits at YOUth. An appointment for the first follow-up visit at 30 weeks of pregnancy (between 29 and 33 weeks’ gestational age) is made after the baseline visit. Around 40 weeks of pregnancy a questionnaire about labour and birth is sent.

#### 4.1.2 ‘Around 0’: 2^nd^ and 3^rd^ follow-up visit

In this measurement wave the babies from the included mothers visit our Child Research Center twice: when they are 4–6 months old and when they are 9–11 months old. At both visits the children are at our center for approximately 5 h. However, only 45 min of actual testing is planned during these days. Infants have ample opportunity to sleep or drink at any time judged necessary by their parents or the researcher. The actual time spent in the center is thus dependent on the breaks the child needs.

**Fig. 3:**
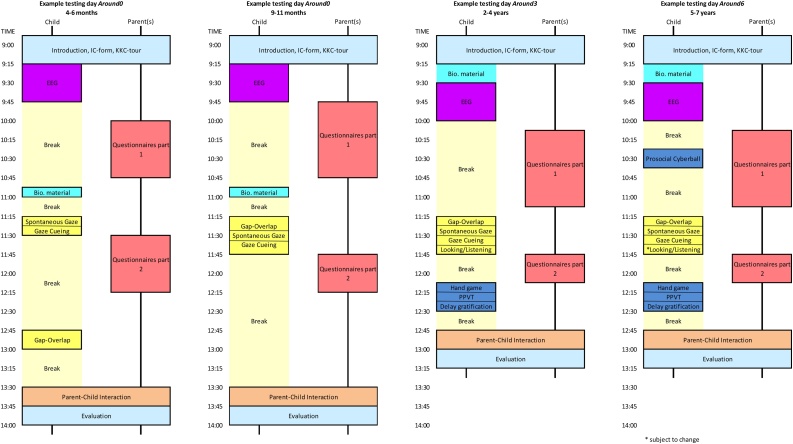
Example test days for YOUth Baby & Child.

Almost the same measurements are performed during the two visits. An example of the testing day can be found in Fig. 3.

#### 4.1.3 ‘Around 3’

When the children reach the age of 2;0–4;11 years old, they are contacted by the Child Research Center for their next follow-up wave. Again, the children are asked to visit our center for a testing day. As the children are older, the total measurement time is longer: approximately 1 h and 15 min. Between the measurements, requests for breaks are honored at any moment. On average the total duration of the testing day is the same as in the previous wave. Again, an example of a testing day can be seen in Fig. 3.

#### 4.1.4 ‘Around 6’

At the time of writing this paper this wave has not been designed in detail yet, but is meant to be a transition between the YOUth Baby & Child cohort and the YOUth Child & Adolescent cohort. The measurements that have been decided upon already are included in the tables, but the measurements in this wave can change after publication of this paper. Children will be between 5;0 and 7;11 years old upon return.

### 4.2 YOUth child & adolescent

In YOUth Child & Adolescent we aim to include around 2,000 children aged 8–10 years old, who are living in Utrecht and its surrounding areas, and their approximately 4,000 parents/caregivers. Recruitment takes place mostly through primary schools. Utrecht and its surrounding areas have approximately 385 primary schools with on average 90 children per school in the appropriate age range. When a school agrees to help YOUth with the recruitment of children, the school first informs the parents, for instance via a newsletter. In this newsletter parents are informed that a YOUth employee will give a presentation for the children about a research day in our center. If parents do not want their children to be in the classroom during the presentation, they can contact the school. After the presentation our employee gives each eligible child a flyer that the child takes home. If parents (and the child) are interested in participating, they can contact the Child Research Center for the information package. Interest can be shown by reply card, through the website, or by telephone or e-mail. If parents and children are interested after reading the brochures, they can contact the Child Research Center again. If the parents do not contact us within two weeks after receiving the information package, the research team contacts the parents to make sure they received the information and asks them if they have any further questions. After a positive response of the parents, the research assistant conducts a brief telephone screening to check for in- and exclusion criteria, and an appointment is made for the testing day. A letter or email is sent to confirm the appointment.

Of all flyers that are handed out to the children, currently 10 % result in a request for an information brochure, of which 60–65 % are included.

#### 4.2.1 ‘Around 9’: baseline visit of YOUth child & adolescent

**Fig. 4:**
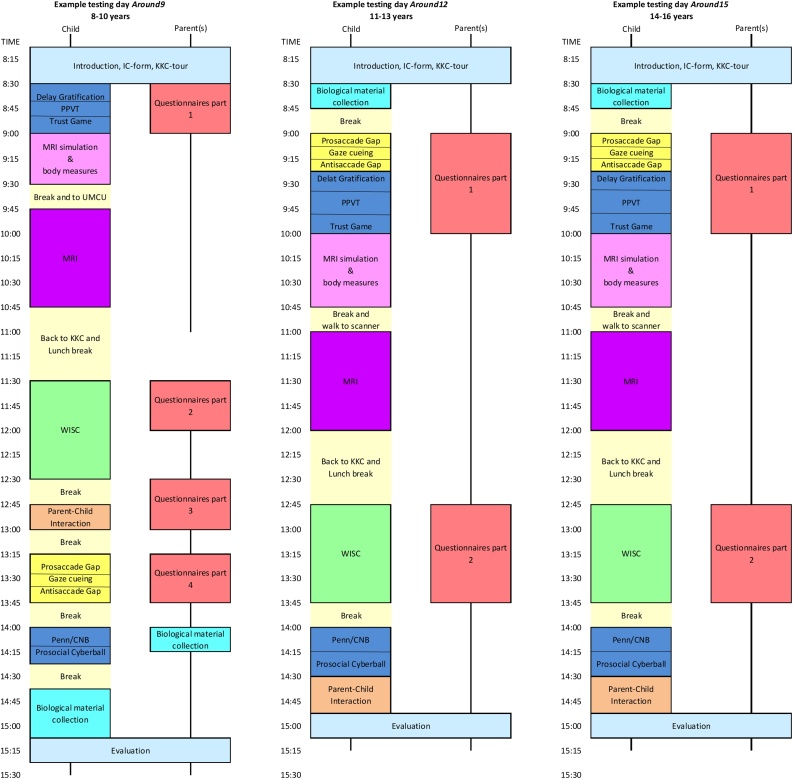
example test days for YOUth Child & Adolescent.

Our baseline measurement wave is called ‘Around 9’ and an example of a testing day can be found in Fig. 4. For this visit the children come to our Child Research Center when they are 8–10 years old. The visit takes approximately seven hours, but the actual testing time is only four and a half hours, due to time taken for breaks. A lunch break halfway the testing day is provided for the children and parents.

#### 4.2.2 ‘Around 12’ and ‘around 15’

Both waves are very similar to ‘Around 9’, both in experiments and in time spent at our center.

Briefly, 3 years after the child visited the center for ‘Around 9’, the parents of the child are contacted by our Child Research Center again for the next wave of follow-up. The duration of the testing day is also the same as in ‘Around 9’, and again a lunch break halfway the testing day is provided. At the time of writing this paper ‘Around 15’ has not been designed in detail yet, but the testing day will be similar to ‘Around 1’2 and the same measures will be taken.

## 5 Measurements

In this section we describe the measurements (experiments, questionnaires, etc) that are used in YOUth. We first describe how measurements were chosen. Then we describe each of the measurements, in the order of the research question as depicted in Fig. 1, i.e. measurements of environmental-, general child- and biological factors, experiments that measure neurocognitive development (both general brain development and neurocognitive development related to the emerging social competence and self-regulation), the main outcome measurements (i.e. social competence and self-regulation) and the long term outcomes.

### 5.1 Choice of measurements

Our aim was to include measurements (i.e. experiments and questionnaires) that have been validated, have a good test-retest reliability, and preferably have been used in other cohorts as well. However, for several determinants and outcomes validated questionnaires are not available. In those circumstances we use questionnaires that are used in other large cohort studies as well, such as the Generation R study, which enables us to collaborate with these cohorts. For all experiments we have the additional requirements that the construct should be usable and measurable longitudinally at multiple ages between 0–18, and that the task is able to tap into critical developmental periods.

For our choice of tasks, we had the following methods available for both cohorts: eye tracking, computer tasks, behavioral tasks. We also had access to ultrasounds and EEG (for YOUth Baby & Child), as well as MRI (for YOUth Child & Teenager). See our appendix 1 for more information about the methods.

### 5.2 Environmental- and general child factors (Table 5)

In YOUth a broad spectrum of environmental determinants are measured, mostly via questionnaires, but also through linkage with registries and through measurements during the testing day. In summary (a more extensive overview is provided in Appendix 2), we obtain information on demographic factors, mental health, lifestyle, stress and life events, personality and personality traits, (social) media use, social networks and peers, sleep, parenting, pubertal development, language development, and daycare use. Furthermore, we ask permission to merge the records of the parents and the children with the data from Statistics Netherlands (CBS). Data on labour and profession, education, income, environment, causes of death, etc. are available in this registry for linkage under strict conditions.

### 5.3 Biological determinants (Table 6)

Table 6 describes the biological determinants that we measure in YOUth. Our biological determinants include both biological materials and body measurements. In YOUth we have a wide collection of biological materials in all different waves to investigate biological determinants of behavioral and brain development.

From all pregnant women and from all children aged 8 years and older, we collect 20 mL serum and 10 mL EDTA-plasma through venapunction, which are stored in 12 aliquots of serum, 6 aliquots of plasma and 3 aliquots of cell pellets for DNA isolation. Plasma and serum are stored only from the pregnant women and children aged 8 years and older, to be able to measure various biomarkers. From all fathers, and from the mothers of the children aged 8 years and older, we collect only a 10 mL EDTA-plasma blood sample, from which 3 aliquots of cell pellets for DNA are stored. No serum is stored for these fathers and mothers as we expect that biomarkers in their blood are not very predictive for the development of these older children. The parents blood samples are only collected at baseline. For the children, we also collect blood samples during follow-up waves.

We also collect a buccal swab sample from all parents at baseline and from all children at baseline and during follow-up waves. Buccal cells are collected with a swab (Sarstedt forensic swab), by gently rubbing and rotating the swab along the inside of the cheek for 5–10 s. DNA can be isolated from these buccal swab samples for later genomic and epigenomic research.

We further collect hair samples from all pregnant women (at 30 weeks of pregnancy) and from all children at each follow-up wave from ‘Around 3′ onwards. As, we expect that most women are not willing to provide more than one hair sample, we chose the later round during pregnancy as we expect that this will provide most information on environmental exposure in the first and second trimester of pregnancy. Approximately 200 strings of hair are cut from the back of the head of the participant as close as possible to the skin. The sample is taped on a sheet of paper and put in an envelope that is sealed and stored for future analysis.

From ‘Around 9’ onwards we ask the children to collect saliva at home 30 min after waking up. Saliva can be used for cortisol and sex-steroid measurements, but potentially for many more biomarkers. Girls that have had their menarche are asked to collect the saliva at the 7th day of their cycle (counting from the first day of menstruation). We have chosen for a fixed day within the cycle to correct for hormonal fluctuations during the cycle. Saliva containers are handed to the children on the testing day. Saliva sample collection requires the participant to spit in this container. The children are asked to send the samples back through normal mail using special envelopes, which are provided by us.

In YOUth Baby & Child, we ask the midwives to draw a 10 mL EDTA-plasma sample from the umbilical cord at birth. At their 30-weeks visit a sample kit is provided to the pregnant women and the midwife receives instructions regarding the cord blood sampling procedures. The samples are sent to the Child Research Center by regular post in a postal etui following the UN3373 regulations.

All biological samples are stored in the central biobank facility of the University Medical Center Utrecht (UMCU). Serum, plasma, and cord blood samples are aliquoted and stored in 900 μL containers at −80 °C freezers for future use. Buccal swabs samples are stored at special containers in the same freezers as well, as are saliva samples. Hair samples are stored in special envelopes in fire proof cabinets in the Child Research Center.

Biomarker research is a rapidly developing field, with new techniques being introduced all the time. Currently, no specific research questions are formulated for the biological materials, other than our general research questions approved by the Medical Research Ethics Committee Utrecht. Therefore, we cannot yet provide details on measurements that will be done with our biosamples other than described above.

Besides collecting body tissues at each round, we also register physical development. In YOUth Baby & Child we use a questionnaire in which the parents copy the child’s height and weight measurements, as well as vaccination status from the books that parents have from their regular visits to the youth health care office. Also, the results from the hearing test of the child are copied from these books to our own records. Then, from ‘Around 3′ onwards, the height (in cm) and weight (in kg) of the child are measured during the testing day in light clothing. Height and weight of the parents is asked in the general questionnaire.

The parents receive online questionnaires on their health and the mother on her periconceptual health and questions regarding the pregnancy and birth. Parents receive a questionnaire regarding the medical health of the child. For specific research questions we ask permission to merge with several databases, such as the databases from the general practitioners (GPs), dentists, pharmacies, youth health registries, etc.

### 5.4 Experiments that measure neurocognitive development

We have developed an extensive neurocognitive development battery in which we measure both general brain development and emerging self-regulation and social competence. The technical details of the measurements are described in appendix 1 and in more detail on our website (https://www.uu.nl/en/research/youth-cohort-study/about-the-youth-cohort-study/youth-data-collection).

#### 5.4.1 Neurocognitive development: general brain development (Table 2)

General brain development is measured with different methods at different waves. A summary of the measurements is shown in Table 2.

**Table 1:** Main outcome measures in YOUth.

	Self-regulation	Social Competence		
Scale	Effortful control	Ages and stages questionnaire-Social Emotional-2	Infant-Toddler Social and Emotional Assessment *	Strength and Difficulties Questionnaire
	IBQ-R-SF/ECBQ-SF/CBQ-SF/ EATQ-R-SF**	ASQ-SE-2	ITSEA	SDQ
Subscale(s)	‘Inhibitory control’ and ‘Attentional focusing/shifting’	‘Communication’ and ‘Interaction with other people’	‘Prosocial behavior’ and ‘Empathy’	‘Prosocial behavior’ and ‘Peer problems’
Measurement waves	All waves parent report on child From ‘Around 9’ onwards EATQ-R-SF child self report is also used	’Around 0’ and ‘Around 3’ parent report on child	’Around 3’ parent report on child	From ‘Around 3’ onwards parent report on child In addition a child self report is used in’ Around 12’ and ‘Around 15’

* Only in part of the children until September 2019.

** IBQ-R-SF: Infant Behavior Questionnaire Revised Short Form; ECBQ-SF: Early Childhood Behavior Questionnaire Short Form; CBQ-SF: Children’s Behavior Questionnaire Short Form; EATQ-R-SF: Early Adolescent Temperament Questionnaire-Revised Short Form.

**Table 2:** Measurements of general brain development in YOUth.

What	How	YOUth Baby & Child				YOUth Child & Adolescent		
		Rzw	R0	R3	R6	R9	R12	R15
Brain anatomy and connectivity	3D fetal ultrasound	X						
	EEG*		X	X	X			
	sMRI**					X	X	X
General cognitive development	WISC***				X	X	X	X
	CNP****					X	X	X
	Linkage with school records and end-of-primary school result						A*****	

* EEG: Electroencephalography.

** sMRI: structural magnetic resonance imaging.

*** WISC: Wechsler Intelligence Scale for Children.

**** CNP: Computerized Neuro-psychological test battery.

***** A: cannot be done before the children leave primary school (usually at the age of 12).

At 20 and 30- weeks gestational age, the pregnant women included in YOUth receive an advanced fetal neurosonogram. This ultrasound takes 20−30 min, depending on the position of the baby. Advanced fetal neurosonography consists of aqcuiring six 3D volume sweeps: two transversal (transthalamic and transcerebellar),two coronal (transthalamic and transcerebellar), a midsagittal and a parasagittal plane. In addition, fetal biometry parameters including head circumference, abdominal circumference, femur length, and a Doppler velocity flow patterns of the umbilical artery, the medial cerebral artery, and the uterine arteries, are measured (Albers et al., 2018; International Society of Ultrasound in, O. and C. Gynecology Education, 2007; Monteagudo and Timor-Tritsch, 2012).

At ‘Around 0’, ‘Around 3’ and ‘Around 6’, general brain development is measured with continuous Electroencephalography (EEG). We record EEG while infants view social and non-social videos. In the social videos women are singing nursery rhymes and in the non-social videos toys are moving by themselves. This design is typically used to examine how differences in frequency bands relate to differences in processing social versus non-social information (Jones et al., 2015). As such, this experiment also belongs to our emerging social competence measurements. At the same time we can use the complete continuous EEG session to provide us with brain connectivity estimates indicative of general brain development and with global frequency-bands.

Development in children’s head circumference is tracked via the records parents keep from their regular visits to the youth health care office that each infant in the Netherlands has.

In ‘Around 9’, ‘Around12’, and ‘Around 15’ brain development is measured using magnetic resonance imaging (sMRI) aimed to measure development of the brain’s structure and functioning during puberty in relation to genes, environment and the development of self-regulation and social competence. Prior to scanning, each child undergoes a practice session in a mock scanner. It has been shown that implementing a mock procedure decreases scanner-related distress in children (Durston et al., 2009). Therefore, a scanner simulation is designed to mimic the actual experience in the scanner (Durston et al., 2009) and determine a proxy of scanner-related distress. At any time, the MRI session can be canceled if the child or the parent/guardian indicates that the child does not feel comfortable continuing and this is explaing throuroughly to parents and child. The YOUth MRI protocol comprises different types of MRI scans acquired on a Philips Ingenia 3.0 T CX scanner: i.e. for brain anatomy T1-weighted images; for white matter integrity diffusion-weighted images (DWI) and for brain activity resting-state and task-based functional MRI [See for more details in this special issue: Buimer et al., 2020].

In ‘Around 9’, ‘Around 12’ and ‘Around 15’, general cognitive development is measured by estimating the child’s intelligence with the Wechsler Intelligence Scale for Children. We started data collection using the third edition(WISC-III latest Dutch version (up to 2018)) (Wechsler, 2003) assessing six subtests of the WISC-III: arithmetic, block design, coding, digit span, similarities and vocabulary. The WISC-V (WISC-V Dutch version) (Wechsler, 2018) is administered from the moment it was made available in 2018. We assess seven subtests of the WISC-V: block design, coding, digit span, figure weights, matrix reasoning, similarities and vocabulary. In ‘Around 6’, the WPPSI/WISC will be used to asses intelligence.

The **Computerized NeuroPsychological Testbattery (CNP)** of the University of Pennsylvania is developed to capture specific cognitive domains that link to functioning of specific brain systems (Gur et al., 2010; Swagerman et al., 2016). At ‘Around 9’, ‘Around 12’, and ‘Around 15’ a subset of 3 tasks from the CNP battery is included: the Mouse Practice Task to ensure the child has sufficient skills to perform the computer tasks, the Penn Word Memory Test (PWMT) to measure episodic memory and the 40-item Emotion Recognition Test (ER-40) to measure emotion recognition. During the PWMT, the child views 20 target words that are subsequently mixed with distractors to test whether the child correctly identifies the targets. This test is repeated after a 20 min delay. In the Emotion Recognition task the child is asked to identify emotions (happy, sad, anger, fear, neutral) in a multiple choice format.

#### 5.4.2 Neurocognitive development: social competence (Table 3)

The neurocognitive development of (underlying skills for) social competence is measured using several tasks with different techniques. Note that YOUth not only measures development in social competence itself, but also captures development in the underlying skills essential for developing good social competence: social encoding, social problem solving, emotion regulation, communication, and empathy [in this issue: (Junge et al., 2020)].

**Table 3:** Measurements of emerging social competence in YOUth.

What	How	YOUth Baby & Child				YOUth Child & Adolescent		
		Rzw	R0	R3	R6	R9	R12	R15
Emotional Face Processing	EEG(ERP)*		X	X	X			
	fMRI**					X	X	X
Gaze cueing experiment	Eye tracking		X	X	X	X	X	X
Face Popout experiment	Eye tracking		X	X	X			
Looking while listening	Eye tracking			X				
Peabody Picture Vocabulatory Task language comprehension	Computer			X	X	X	X	X
Trust game reciprocity	Computer					X	X	X
Prosocial cyberball empathy	Computer					X	X	X
Parent Child Interactions	Video		X	X	X	X	X	X
Interpersonal Reactivity Index (IRI) (De Corte et al., 2007; Davis, 1980)	Questionnaire***			PC	PC	CH	CH	CH

ᵃ Parent child report; ᵇ Child self report.

* ERP: Event-Related Potential.

** fMRI: functional magnetic resonance imaging.

*** PC and CH define who completes the questionnaire. PC; Parent report on child; CH: Child self report.

**(Emotional) Face processing:** Recognizing possible interaction partners as well as differentiating between emotional facial expressions are considered vital skills underlying social competence (Junge et al., 2020). At YOUth Baby & Child (‘Around 0’, ‘Around 3’, and ‘Around 6’) we use EEG (event-related potentials, more specifically) to measure whether infants can tell apart faces from houses, and whether they can discriminate between different facial epressions. The 5-months-olds passively see only neutral faces and houses while the 10-month-olds and older children in ‘Around 3’ additionally see fearful or happy faces (van der Velde and Junge, 2020).

For the YOUth Child & Adolescent waves (‘Around 9’, ‘Around 12’, and ‘Around 15’), we administer the same tasks, but with MRI. While in the MRI scanner, children are asked to passively view (emotional) faces or houses, while their brain activity is recorded. We use exactly the same stimuli and comparisons as in our EEG tasks that is used in YOUth Baby & Child (neutral faces, fearful faces, happy faces, houses) (van der Velde and Junge, 2020).

The **social vs. non-social clips task** (Petros et al., 2005) is also an EEG task designed to capture social competence and tested in all waves of the YOUth Baby & Child cohort, that is within ‘Around 0’, ‘Around 3’ and ‘Around 6’. For more details, see its description in general brain development.

The **gaze cueing experiment** (Friesen and Kingstone, 1998; Frischen et al., 2007; Hessels and Hooge, 2019) is an eye-tracking experiment administered at all waves (‘Around 0’, ‘Around 3’, ‘Around 6’, ‘Around 9’, ‘Around 12’, and ‘Around 15’). It investigates a key component of the mechanism of joint attention, that is, the participant’s ability to follow people’s gaze direction. In the experiment, a face is first presented with direct and then averted gaze. It has been shown that such shifts of gaze direction trigger a reflexive shift of visual attention in the observer. The gaze cue can be both congruent (50 %, meaning that the target appears in the gaze direction of the face) or incongruent (50 %, target in opposite direction from gaze direction). The difference in reaction time between the congruent and incongruent trials is indicative of the strength of reflexive orienting to gaze direction and is our dependent variable.

The **face pop-out experiment** is conducted at ‘Around 0’, ‘Around 3’, and ‘Around 6’. In this experiment, infants view a circular array of five items, with each array containing one face plus four distracters (e.g., bird, car, telephone). Using eye tracking, we examine individual differences in spontaneous gaze behavior and the time it takes before a child focuses on a human face (Hessels and Hooge, 2019; Gliga et al., 2009; Elsabbagh et al., 2013a; Wass et al., 2015).

The **Looking-while-listening task** is measured at ‘Around 3’. It is a 5-minute experiment that measures dynamic language comprehension (Fernald et al., 2008). During this task children see two familiar objects at a time, for example a ball and a shoe. Then the children are asked to look at one of these objects (e.g., “where is the ball?”). Using eye tracking, we examine individual differences both in accuracy of word comprehension (duration of looking at the correct object from word onset) and in speed of word recognition (the time between looking from the distractor object (e.g. the shoe) to the target object (e.g. the ball)).

The **Peabody-Picture vocabulary task** (PPVT (Dunn and Dunn, 1981); for Dutch, the PPVT-III-NL (Dunn and Dunn, 2005)) is a computer task and administered at ‘Around 3’, ‘Around 6’, ‘Around 9’, ‘Around 12’ and ‘Around 15’. This is a widely used task to evaluate a participant’s vocabulary size for his or her age, and serves as a proxy of general language performance. It is normed for participants up to 90 years of age. Participants see on each trial an array of four pictures and hear a word that matches one of these four. They need to click or point to the correct picture. There are in total 204 trials, presented in sets of 12 trials. Participants start with the set that is age-appropriate for them. They stop once they make more than six errors in a set of 12 trials. Total number of trials is thus dependent on the participant’s vocabulary knowledge, and the task should last approximately 10 min.

**(Developmental) Trust-game** (Berg et al., 1995; Crone and Güroğlu, 2013). The trust game measures reciprocity, which is crucial for maintaining positive interactions, and is administered at ‘Around 9’, ‘Around 12’, and ‘Around 15’. In this game, each wave consists of two players, who take turns in dividing a sum of money. The first player (either the child or each wave a simulated novel player) gets two options on how to divide a sum of money: make a pre-defined selection or let player 2 decide how to distribute the money (i.e. player 1 trusts player 2 with the money), in which case the stakes are tripled. Player 2 subsequently receives two options of how to distribute the money: either both players end up with a fair share or player 2 keeps everything. To focus on reciprocity (i.e. willingness to return favors), the participant always starts as player 2 (block design). Developmental studies with this paradigm show that reciprocity increases with age (van den Bos et al., 2010). Trial manipulations are whether the pre-defined distribution for Player 1 at the first stage is relatively high or low compared to what the other player receives and whether the stakes at the start are small or large. Participants are told that they will receive the money from a few waves that is randomly sampled from all waves (van den Bos et al., 2010). However, each participant receives a fixed amount of €1,50.

**Prosocial cyberball** (Riem et al., 2013) is a computer task and is measured at ‘Around 9’, ‘Around 12’ and ‘Around 15’. This task reflects a component of social competence, that is, children’s empathy and their ability to act accordingly. In the prosocial cyberball task, children play a ball-tossing game on-line with three other children (computer-simulations of peers, same gender, with typical Dutch names). In the first block (48 trials) all children receive the ball on average every fourth throw. If the participant receives the ball, he/she can decide who is next to receive the ball. In the second block (48 trials), one of the three peers is systematically ignored (i.e., never receives the ball from the other two peers). Participants can however show empathy by compensating: when they receive the ball, they might choose to throw the ball more often to the ignored player. The DV is the increase in throws to the ignored player in this block relative to first block. This version of prosocial cyberball task has been administered from the age of five years in Generation-R cohort study (Jaddoe et al., 2006).

A **parent-child interaction (PCI) session** is included to assess how the development of social competence and self-regulation are shaped in the context of interactions with the social environment, especially the parents [(Karreman et al., 2006) Dekovic, 2006]. Each PCI session takes about 15-minute. In YOUth Baby & Child (‘Around 0’, ‘Around 3, and ‘Around 6’) the PCI includes free play and two age appropriate structured tasks. For example, reading a book with their child, or teaching the child to complete a puzzle. Depending on their age the child is seated in a baby bouncer or on a rug on the ground next to the parent. In YOUth Child & Adolescent, parent and child are asked to discuss a difficult and a pleasant topic. First, they are instructed to discuss a conflict they had the previous month (i.e., about home work; manners; amount of TV or computer games; see (Granic et al., 2003)). Next, they discuss plans on where to go for a short break. During the discussions, the parent and child are alone in the room and seated on chairs. The interaction is videotaped with consent of the parent (and of the children at older ages).

The **Interpersonal reactivity index** (IRI) (De Corte et al., 2007; Davis, 1980) is a questionnaire that measures empathic tendencies and consists of four subscales, Empathic Concern (sympathy for others in need), Perspective Taking (considering for others’ viewpoint), Fantasy (identifying with fictional characters in books and films), and Personal Distress (self-oriented, negative arousal in response to others' distress). In YOUth, only Empathic Concern and Perspective Taking are used as a measure of social competence in ‘Around 3’, ‘Around 6’, ‘Around 9’, ‘Around 12’, and ‘Around 15’. We use a parent report on the child in ‘Around 3’ and ‘Around 6’; from ‘Around 9’ onwards we use the children’s self report.

#### 5.4.3 Neurocognitive development: self-regulation (Table 4)

Similar to social competence, the neurocognitive development of self-regulation is also measured using several tasks with different techniques.

The **prosaccade gap-overlap experiment** (Saslow, 1967; Elsabbagh et al., 2013b; Van der Stigchel et al., 2017) is an eye-tracking experiment used to measure attentional disengagement from a central stimulus in order to shift gaze direction to a peripheral stimulus. The experiment is used in ‘Around 0’, ‘Around 3’, ‘Around 6’, ‘Around 9’, ‘Around 12’, and ‘Around 15’. The experiment contains three conditions; i) Gap, in which the central stimulus disappears 200 ms before the appearance of the peripheral target; ii) Baseline, in which the central stimulus disappears simultaneously with the appearance of the peripheral target; iii) Overlap, in which the central stimulus remains on screen during peripheral target presentation. Attentional disengagement is defined as the difference in saccadic reaction time between gap and overlap conditions .

**Table 4:** Measurements of emerging self-regulation in YOUth.

What	How	YOUth Baby & Child				YOUth Child & Adolescent		
		Rzw	R0	R3	R6	R9	R12	R15
Prosaccade gap-overlap experiment	Eye tracking		X	X	X	X	X	X
Delay gratification task	Video					X	X	X
Hand game task	Video							
Stop signal anticipation task inhibition	fMRI					X	X	X
Antisaccade gap-overlap task experiment	Eye tracking					X	X	X

**Table 5:** Environmental determinants measured in YOUth.

What	How	YOUth Baby & Child				YOUth Child & Adolescent		
		Rzw	R0	R3	R6	R9	R12	R15
Demography	Demography questionnaire*	MO/FA	MO/FA	MO/FA	MO/FA	MO/FA	MO/FA	MO/FA
	Work and Work environment questionnaire*	MO/FA	MO/FA	MO/FA	MO/FA	MO/FA	MO/FA	MO/FA
	Merging with CBS							
Mental health	Psychiatric family history questionnaire*	MO/FA	MO/FA	MO/FA	MO/FA	MO/FA	MO/FA	MO/FA
	Adult self report questionnaire (ASR) (Achenbach and Rescorla, 2003)*	MO/FA		MO/FA	MO/FA	MO/FA	MO/FA	MO/FA
	Social Responsiveness Scale questionnaire (SRS-A) (Constantino and Gruber, 2005; Noens et al., 2012) *	MO/FA				MO/FA		
	Strengths and Weaknesses of Attention-Deficit/Hyperactivity-symptoms and Normal-behaviors rating scale questionnaire (SWAN rating scale) (Polderman et al., 2007; [Bibr bib0025][Bibr bib0425])*					PC		
	Child Behavior Checklist questionnaire (CBCL) (Verhulst et al., 1996a; Verhulst et al., 1996b)*			PC	PC	PC	PC	PC
	Teacher Report Form (TRF) (Verhulst et al., 1997)*					TC		
	Brief Symptom Inventory questionnaire (BSI) (De Beurs, 2006; Derogatis and Melisaratos, 1983)*	MO/FA						
	Edinburgh Postnatal Depression Scale questionnaire (EPDS) (Pop et al., 1992; Cox et al., 1987) *		MO					
Life style	(Pre)pregnancy life style questionnaire*	MO/FA				MO/FA		
	General parental lifestyle questionnaire*	MO/FA	MO/FA	MO/FA	MO/FA	MO/FA	MO/FA	MO/FA
	Smoking and substance (ab)use questionnaire*					CH	CH	CH
	Parental smoking and substance (ab)use questionnaire*	MO/FA	MO/FA	MO/FA	MO/FA	MO/FA	MO/FA	MO/FA
Nutrition	Nutrition questionnaire*		PC	PC	PC	PC	CH	CH
Media	Food intake during pregnancy questionnaire (Health et al., 2018)*	MO						
	Physical Activity Questionnaire (PAQ) questionnaire (Bervoets et al., 2014; [Bibr bib0420][Bibr bib0410][Bibr bib0415])*				CH^a^	CH^ab^	CH^b^	CH^b^
	Short QUestionnaire to ASsess Health enhancing physical activity (SQUASH) questionnaire (Wendel-Vos et al., 2003)*	MO						
	Sports and hobbies questionnaire*			PC				
Stress and life events	Major Life Events questionnaire*	MO/FA	MO/FA	MO/FA	MO/FA	MO/FA	MO/FA	MO/FA
	Social Readjustment Rating Scale and Lijst met langdurig belastende omstandigheden questionnaire (Hendriks et al., 1990)*	MO			MO/FA	MO/FA	MO/FA	MO/FA
	Childhood memories questionnaire (Arrindell et al., 1986)*	MO/FA				MO/FA		
	Childhood Trauma Questionnaire (CTQ) and Childhood memories (Bernstein et al., 1994)*	MO/FA				MO/FA		
Personality and personality traits	Utrechtse Coping Lijst questionnaire (UCL) (Schreurs et al., 1993)*	MO/FA			MO/FA	MO/FA	MO/FA	MO/FA
	NEO-Five-Factor Inventory-3 questionnaire (NEO-FFI-3) (Costa and MacCrae, 1992)*	MO/FA				MO/FA		
	Portrait Values Questionnaire - Revised (PVQ-RR) (Schwartz et al., 2012)*	MO/FA				MO/FA		
	Self-Perception Profile for Adolescents “Competentie Belevingsschaal” questionnaire (CBS)) (Treffers et al., 2004; [Bibr bib0730][Bibr bib0735])}*					CH	CH	
	Barrat Impulsiveness Scale-Brief questionnaire (BIS-Brief) (Patton et al., 1995; [Bibr bib0575][Bibr bib0585])*					CH	CH	
	Gender identity questionnaire (GI)*			PC	PC	PC	PC	PC
	Quick Big Five questionnaire (QBF) (Vermulst and Gerris, 2009)*				PC	PC	PC	PC
(Social)media	Fiction questionnaire (FVL) (Fikkers et al., 2013)*			PC	PC	CH	CH	CH
	Media education questionnaire (Valkenburg et al., 2013; Valkenburg et al., 1999)*				PC	PC	PC	PC
Social networks/Peers	Network of relationships Social Provision Version - Short Form questionnaire (NRI-SPV-SF) (Furman and Buhrmester, 1985; [Bibr bib0145][Bibr bib0255])*					PC/CH	PC/CH	PC/CH
	Bullying questionnaire*				PC	PC	PC	PC
	Social Support List questionnaire (SSL) (Bridges et al., 2002; van Sonderen, 1997) *		MO					
Sleep	Sleep Self Report questionnaire (SSR) (van Litsenburg et al., 2010)*					CH		
	Promis Sleep Item Bank questionnaire (Haverman et al., 2016; van Kooten et al., 2018) *						CH	CH
	Pittsburgh Sleep Quality Index questionnaire (PSQI) (Buysse et al., 1989)*	MO						
	Children's Sleep Habits Questionnaire (CSHQ) (van Litsenburg et al., 2010; Owens et al., 2000)*				PC			
Parenting	Parental Control Scale questionnaire (PCS) (Barber, 2002; [Bibr bib0040][Bibr bib0050])*					CH	CH	CH
	Child Report of Parenting Behavior Inventory questionnaire (CRPBI) (Schaefer, 1965a)*					CH	CH	CH
	Alabama Parenting Questionnaire (APQ) (Shelton et al., 1996; [Bibr bib0235][Bibr bib0225][Bibr bib0665][Bibr bib0200])*					PC	PC	PC
	Child‐Rearing Questionnaire (NOV) (Gerris et al., 1993)*					PC	PC	PC
	Parenting Dimensions Inventory questionnaire (PDI) (Slater and Power, 1987)*					PC	PC	PC
	Parenting stress index (Nijmeegse Ouderlijke Stress Index, NOSI) (De Brock et al., 1992; Abidin, 1983)*		PC	PC	PC	PC	PC	PC
	Vragenlijst Toezicht Houden(VTH) (Parental Monitoring Questionnaire) (Dekovic, 1996)*						PC	PC
	Parenting Practices questionnaire (PP):Brown, 1993; Kerr, 2000; Stattin, 2000; Keijsers, 2009}*						CH	CH
	Comprehensive Early Childhood Parenting Questionnaire (CECPAQ) (Verhoeven et al., 2017)*		PC	PC	PC			
Pubertal development	Pubertal development scale questionnaire (PDS) (Carskadon and Acebo, 1993) *					CH	CH	CH
	Sexual development questionnaire (Van de Bongardt et al., 2013; [Bibr bib0035][Bibr bib0165][Bibr bib0150]) *						CH	CH
Language	Language situation questionnaire*		PC	PC	PC	PC	PC	PC
	Clinical Evaluation of Language Fundamentals questionnaire (CELF){wiig, 2004; Wiig, 2012; Kort, 2008*			PC^c^	PC ^d^	PC ^d^	PC ^d^	PC ^d^
	Communicative Development Inventory questionnaire (N-CDI) (Zink and Lejaegere, 2020) *		PC^e^	PC^ef^				
Daycare	Daycare questionnaire*		PC	PC	PC			

* MO, FA, PC, TC, CH define who completes the questionnaire. MO: Mother self report; FA: Father self report; PC; Parent report on child; TC: Teacher report on child; CH: Child self report; ^a^ PAQ-C; ^b^ PAQ-A; ^c^ PRE-CELF-NL-2 subscale Pragmatics; ^d^ CELF-IV-NL subscale Pragmatics; ^e^ N-CDI-1; ^f^ combination of N-CDI-2 and N-CDI-3.

**Table 6:** Biological determinants measured in YOUth.

What	How	YOUth Baby & Child				YOUth Child & Adolescent		
		Rzw	R0	R3	R6	R9	R12	R15
DNA	Blood parents	X				X		
	Buccal swab parent	X				X		
	Buccal swab Child		X	X	X	X	X	X
	Blood child	X (cord)^a^				X	X	X
Serum/plasma	Blood	X (M)^b^				X	X	X
Hormones	Saliva Child				X	X	X	X
Stress, drugs	Hair	X (M)^b^	X	X	X	X	X	X
Physical health	Periconceptual health questionnaire*	MO/FA				MO/FA		
	Obstetric outcome questionnaire*	PC				PC		
	General health questionnaire*		PC	PC	PC	PC	PC	PC
	General parental health questionnaire*	MO/FA	MO/FA			MO/FA	MO/FA	MO/FA
	Medical family history questionnaire*	MO/FA				MO/FA		
	Anthropometry and vaccinations questionnaire*		PC	PC	PC			
	Anthropometry during testing day			X	X	X	X	X
	Merging records^c^							

a Cord means cordblood.

b M refers to the fact that this was only collected in the mothers at YOUth Baby & Child, whereas DNA was also collected from blood from the fathers and the mothers in YOUth Child & Adolescent. Serum and plasma was not stored for the fathers, nor was a hair sample stored.

c Records are not merged each measurement wave, but only when a specific research questions includes data from these records.

* MO, FA, PC define who completes the questionnaire. MO: Mother self report; FA: Father self report; PC; Parent report on child.

The **antisaccade gap-overlap experiment** (Everling and Fischer, 1998; Munoz and Everling, 2004) is nearly identical to the prosaccade gap-overlap experiment, except for the instruction given to the participants. Children are instructed to look at the opposite side from where the peripheral stimulus appears. As the experiment requires instructions, it is used in ‘Around 6’, ‘Around 9’, ‘Around 12’ and ‘Around 15’. Key variables are the amount of errors made and the saccadic reaction time. It provides a crucial measure of attentional inhibition.

The **gift delay task**, a delay gratification task, is a measure of the child’s self-control (Kim et al., 2013; Kochanska and Knaack, 2003; Kochanska et al., 2001; Kochanska et al., 2000). It is a video task and is measured at ‘Around 3’. During this task the child is seated at a table and receives a present in a gift bag from the research assistant. The research assistant then tells the child he/she forgot to tie a ribbon to the present, and asks the child to wait for the research assistant’s return before opening the present. The research assistant then leaves the room for 3 min, while the parent remains in the same room as the child. The video records of the child’s behavior are post-coded by trained coders (e.g. does the child touch the present, is the present opened).

Delay gratification reflects the capacity to wait for a reward over choosing a smaller immediate reward. The **Delay Gratification Task (DGT)** is a computer task adapted from Richards (Richards et al., 1999; Prencipe et al., 2011; Olson et al., 2009; Isen et al., 2014; Peper et al., 2013). It is administred at ‘Around 9’, ‘Around 12’, and ‘Around 15’. The children are given a series of option between a variable immediate monetary reward and 10 Euros after a certain delay. The delay of the 10 Euro reward varies between 2, 30, 180, or 365 days. Each trail starts with the question if they rather have a specific immediate reward now or 10 Euros after a specific delay. Based on the choices of the participant, the task determines an indifference point per delay. That is, when the immediate reward has the same subjective value as the 10 Euros at that delay. The different delays are presented in random order, as are the immediate rewards. Based on the decision of the child, the immediate reward is adapted on the next trial of that specific delay following a mathematical model described by Richards and colleagues until the indifference point is reached (Richards et al., 1999). The total number of trials depends on the behavior of the participant.

Luria’s **hand game task** was originally used to measure inhibitory control deficits in adults with frontal lesions (Luria et al., 1964). Later the task has been adapted to use it with children (Hughes, 1996). We use this adapted version of the hand game. It is a video task which is measured at ‘Around 3’. During this task, the child is asked to place a flattened hand on the table whenever the researcher presents a fist and to present a fist whenever the researcher places a flattened hand on the table. Each child is first seated at a table with the researcher and is asked to mimic the researcher as he/she presents a fist and flattened hand on the table in front of the child. This is be done in order to demonstrate that the child possesses the ability to manipulate his or her hand into these shapes. The child is then taught the instructions of the task. The child has at least two practice trials during which he or she is praised or corrected. After that 16 test trials is administered, eight with the experimenter’s fist as the stimulus, and eight with the experimenter’s flattened hand as a stimulus, arranged in a fixed pseudorandom order. The percentage correct trials is calculated.

##### 5.4.3.1 Stop Signal Anticipation functional MRI (fMRI) task

While in the MRI scanner, children are asked to perform the Stop Signal Anticipation task while brain activity is recorded. It is measured at ‘Around 9’, ‘Around 12’, and ‘Around 15’. This task is adopted from Vink and colleagues (Vink et al., 2005; Zandbelt and Vink, 2010) and measures the developmental neural mechanisms underlying reactive inhibition (outright stopping) and proactive inhibition (anticipation of stopping). Response inhibition is considered an important aspect of self-regulation. Children are instructed to stop a moving bar at a specific location (go trails) by pressing a specific response box button. In some trials, the bar stops moving (stop signal) and the participants need to inhibit their response. A cue at the beginning of the trial indicates the probability that the bar will stop (green bar = 0%, orange bar = 17 %, red bar = 33 %). The onset of the stop signal varies from one trial to the next according to a staircase procedure that is dependent on the participant’s response time (Zandbelt et al., 2008). The task lasts 10 min and to ensure that the children understand the task, they are trained in the MRI simulation scanner before the scanning procedure starts.

### 5.5 Primary outcome measurements

As stated in the introduction, the main outcome measures in YOUth are social competence and self-regulation, skills that are essential for functioning in society and for reducing risk of behavioral and emotional problems. In YOUth the primary outcome measures of development of social competence and self-regulation are questionnaires (see Table 1).

**Social competence** is measured in babies and toddlers using the complete parent proxy-report of the Ages and Stages Questionnaire: Social-Emotional, Second Edition (ASQ-SE-2), from which we consider the subscales ‘social communication’ and ‘interaction’ as most relevant (Squires et al., 2002; Steenis et al., 2015). At the start of ‘Around 3′ we measured the subscales ‘prosocial behavior’ and ‘empathy’ of the Infant-Toddler Social Emotional Assessment (ITSEA) (Visser et al., 2010; Carter et al., 2003; Briggs-Gowan and Carter, 1998). However, the ITSEA cannot be used over the complete age range of this wave. We, therefore, amended our protocol and use the subscales’Prosocial behavior’ and ‘Peer problems’ of the Dutch version of the Strengths and Difficulties Questionnaire - Parent Form for children (SDQ – subscales ‘Prosocial behavior’ and ‘Peer problems’) (van Widenfelt et al., 2003; Goodman, 1997) from ‘Around 3’ onwards in stead (the ITSEA was measured in 129 children only). In addition, in the measurement waves ‘Around 12’ and ‘Around 15’ the full-scale child self report versions of the SDQ (van Widenfelt et al., 2003; Goodman et al., 1998) are used.

**Self-regulation** is measured with the Dutch versions of Mary Rothbarth’s Temperament Questionnaires, which is a set of age-specific questionnaires to measure temperament.In YOUth Baby & Child only the subscales Perceptual Sensitivity, Low Intensity Pleasure, Attentional Focusing, and Inhibitory Control are assessed. Additionally, in ‘Around 6’ the subscale Impulsivity is also assessed. At ‘Around 0’ we use the Infant Behavior Questionnaire Revised Short Form for parents (IBQ-R-SF: translated by M. Roest-de Zeeuw and K. van Doesum) (Putnam et al., 2014). At ‘Around 3’ we use the Early Childhood Behavior Questionnaire Short Form for parents or the Children’s Behavior Questionnaire Short Form for parents (ECBQ-SF: translated by R. de Kruif, T. Willekens and L. de Schuymer (Putnam and Rothbart, 2006) or CBQ-SF: translated by M. Majdanzic) (Putnam and Rothbart, 2006). At ‘Around 6’ we also use the CBQ-SF for parents. In all waves of YOUth Child & Adolescent, the full-scale Early Adolescent Temperament Questionnaire-Revised Short Form for parents (EATQ-R-SF: translated in Dutch by C.A. Hartman) is used (Ellis and Rothbart, 2001). In addition, the subscales Inhibitory Control and Attention of the EATQ-R children’s self report are assessed in all waves of YOUth Child & Adolescent (Ellis and Rothbart, 2001).

### 5.6 Long-term outcomes

Long term outcomes include school achievements, psychosocial problems, psychiatric disorders, etc.

Psychiatric disorders are measured by the Child Behavior Check List (CBCL), which is measured at each wave from the age of 2 and older (1.5–5 years: (Verhulst et al., 1996a); 4–18 years: (Verhulst et al., 1996b)). At the start of ‘Around 9′ we asked the teachers of the participating children to fill in the Teachers’s Report Form (TRF) (Verhulst et al., 1997). However, during our yearly progress report, the response of the teachers appeared to be extremely low (below 50 % response). We therefore decided to terminate the TRF before completion of the wave.

All other long term outcomes can be obtained through linkage with several databases. For instance, we ask parents permission (and children from the age of 12 and older) to merge their YOUth data with several databases, including the school records, general practitioner records, the psychiatric case registry Middle-Netherlands, etc. We also ask permission to merge with the “End of Primary Education Test”. Each child in the Netherlands takes such a central test, which is obligatory and aims to predict the best secondary school level of a child at the end of its primary school.

Linkage with these registries will not be done automatically, but only when researchers using our data have specific research questions that require linkage with these databases and have the funding to do so. Approximately 93 % pf the parents give permission for linkage to registries for themselves and 96 % give permission to link information of the children to these registries. There is some variation between the registries with the least objections for linkage with Statistics Netherlands, denstist, hospital records and hospital records and the most objections for linkage with the health insurance companies (approximately 10 % refuse).

## 6 Data quality and management

Before YOUth staff is allowed to perform measurements on participants, they are trained and monitored centrally. All measurements are under supervision of staff members with experience in that field of research and the specific measurement technique involved. These staff members regularly check the quality of the data. Before the start of a measurement wave all measurements are tested in small pilot studies. All YOUth data are collected and stored according to several principals. Data should be FAIR (Findable, Accessible, Interoperable and Re-usable), it must be possible to share data with internal and external partners, all data must be secured against theft, loss and damage, data cannot be changed and should be available not only during the study but also afterwards. Details on data collection, secure storage and handling are described in detail in another paper in this issue [In this issue: Zondergeld et al., 2020]

YOUth stimulates the actual use of the data as broadly as possible, to answer as many specific research questions as possible. For the specific procedures and rules on how to apply for data, we refer to our website: https://www.uu.nl/en/research/youth-cohort-study/data-access.

## 7 Current status

At the time of writing this paper approximately 2,100 pregnant women are included, over 1,400 babies of 5 months old visited our center, 1,200 babies of 10 months old, and 100 toddlers (aged 2, 3, or 4 years old). In YOUth Child & Adolescent we included already over 1,300 children and their parents. At the time of writing this paper we cannot provide information on the retention rate in this cohort. Based on other large cohort studies we expect that in each round we will have a drop-out rate of 20 %. Based on the numbers now in YOUth Baby & Child (which should be interpreted with some caution as recruitment is still ongoing), it seems that this was a conservative estimate as almost 90 % of the mothers return with their babies at “Around 0”, and of those 97 % return at “Around 3”, although the number of included toddlers in that group is still very small. We are currently working on an online instrument on our website that keeps track of these numbers.

## 8 Strengths and limitations

Although several birth- and child cohorts exist in the Netherlands and other countries, very few exist with a focus on behavioral and brain development. For birth cohorts, only the FinnBrain birth cohort study measures the effects of prenatal exposure on child (brain) development and has brain development measures at young ages (Karlsson et al., 2017). The Environmental influences on Child Health Outcomes (ECHO) study aims to investigate the impact of environmental influences on child development. It has a very broad scope and studies five key outcome domains, one of which is neurodevelopment, which measures attention, emotions, intelligence, and behavior (Forrest et al., 2018). ECHO has some overlap with the measurements in YOUth, both in biomaterials, of which they have a very extensive collection, and in neurodevelopmental measurements, but with limited imaging. Generation R is a large Dutch birth cohort (Jaddoe et al., 2010) that started in 2001, where general brain development was measured, using MRI, at ages 7, 9, and 13. No measures of brain development at younger ages are available. However, in 2017 Generation R *Next* started, a new birth cohort in the Netherlands with a focus on brain development. This initiative provides very good opportunities for collaboration.

With respect to the child cohorts including older children, in the Netherlands the BrainScale study investigates the influences of genes and environment on cognitive brain development throughout adolescence in twin families (Teeuw et al., 2019). Internationally, the Adolescent Brain Cognitive Development Study (ABCD) investigates determinants of brain development in children aged 9 years and older, and currently includes almost 12,000 children (Garavan et al., 2018). In this study a large test-battery is administered that broadly measures brain and behavioral development in children, and thus overlaps to a large extent with YOUth, enabling replication and collaboration. In addition, YOUth has a strong focus on social competence and self-regulation. The IMAGEN study is another large cohort, that included 2,000 children at age 14 with a follow-up measurement at age 16–18 years (Schumann et al., 2010). Although this study includes older children than YOUth, the measurements of structural and functional brain imaging largely overlaps with YOUth. So, this study is also very well suited for replication and collaboration.

YOUth is unique in its multidisciplinary collaboration. Researchers with many different expertises (i.e. medical doctors, psychologists, neuropsychologists, behavioral scientists, researchers with expertise on (social) media use, language researchers, statisticians, epidemiologists, animal scientists, etc) and from many different disciplines (Social and Behavioral Sciences, Medicine, Veterinary Sciences, Humanities, and Science) are working together to create this cohort and use its data to its full potential.

Another strength of this study is the broad range of determinants and intermediate outcomes that are measured, resulting in a very large infrastructure for future studies on behavioral development in children and the intermediating role of neurocognitive development.

YOUth uses an accelerated longitudinal design and measure broad age ranges. As stated previously, this design enables us to span the complete age range in a shorter amount of time and will provide detailed information on development over time.

However, we should also acknowledge some limitations of the study. First, even though we have ample power to study our continuous main outcomes (self-regulation and social competence) we have little power for some of the long-term outcomes, such as general functioning outcomes, including psychosocial functioning, academic achievements, problem behavior, and psychiatric disorders. The incidence rates of these outcomes differ greatly (ranging approximately from 1% to 25 %). Therefore, the power to study these outcomes will depend on the amount of cases that will arise in this cohort.

Currently, in the population that has been included so far, we find an overrepresentation of subjects with a high socio-economic status (SES). It is a well-known phenomenon that in population-based studies low SES populations are underrepresented (Galea and Tracy, 2007). This is problematic as this could lead to both non-generalizability and bias in our results. Therefore, we recently invested extra effort and money to increase the inclusion rates in low SES populations.

Finally, we are unable to perform non-response analyses, as we have no information on the characteristics of all eligible pregnant women and children.

## 9 Embedding

YOUth is part of the Consortium on Individual Development (CID), which is granted in research funding by the Ministry of Education, Culture and Science as part of the Gravitation Program. With the Gravitation Program, the Dutch Government aims to encourage research by consortia of top researchers in the Netherlands. CID aims to understand and predict how the interplay of child characteristics and environmental factors results in individual differences in the development of social competence and self-regulation of the child. The YOUth cohort is set up as part of the first work package of this consortium, to help answer this question. Generation R is also part of CID, which, as mentioned above, facilitates collaboration greatly.

YOUth is also part of the strategic theme “Dynamics of Youth’ of Utrecht University, which combines excellent child research from all seven faculties. Researchers from different disciplines integrate their expertise to answer crucial questions for future generations. Utrecht University is a pioneer when it comes to interdisciplinary cooperation. Furthermore, YOUth is part of the UMC Utrecht Brain Center.

## Grant support

The YOUth cohort is part of the Consortium on Individual Development (CID), which is funded through the Gravitation program of the Dutch Ministry of Education, Culture, and Science and the Netherlands Organization for Scientific Research (NWO grant number 024.001.003). (https://www.uu.nl/en/research/dynamics-of-youth/youth).

## Declaration of Competing Interest

The authors report no declarations of interest.
