# Supplementary material for: The YOUth study: Rationale, design, and study procedures
Source: Dev Cogn Neurosci. 2020 Oct 7;46:100868. doi: 10.1016/j.dcn.2020.100868 (PMC7575850; doi:10.1016/j.dcn.2020.100868)
Supplement: Supplementary file 1 [file mmc1.docx]

**Appendix 1**

**Techniques used**

The neurocognitive battery uses several techniques to conduct all the tasks and experiments:

1. Ultrasounds: The ultrasounds are performed by trained sonographists using a Voluson E10 Ultrasound (GE Healthcare). Using a regular abdominal probe several standardized images are obtained and saved according to study protocol[1].
2. EEG: For all EEG measurements we use the BIOSEMI recording system with 32 electrodes integrated in a cap configured to the standard International 10-20 System. Continuous EEG was acquired at a 2048Hz sample rate using Actiview (version 7.05)(BioSemi, Netherlands). Caps for different head sizes are available and the individual cap size of the child is determined by measuring the circumference of the child’s head (over inion, nasion and both preauricular points) using a flexible measuring tape. The experimenter then carefully puts the cap on the head of the child and inserts conductance gel in the cap to decrease resistance from the outer layers of the skull to improve the measurements. Additionally, one flat-typed electrode is placed below the left eye in line with FP1 on the cap (to measure eye blinks). Tasks start when electrodes offset are less than 20 μv. Children sit (either on parent’s lap or in a highchair) in a semi-dark room, facing at 65 cm distance a 23-inch monitor on which the visual stimuli are presented. To co-register whether children attend to the visual stimuli, we record their looking behavior with a web-cam. More details about the procedure can be found elsewhere [2].

1. Eye tracking: All eye-tracking experiments are conducted using the Tobii TX300 eye-tracker (Tobii TX300, Tobii Technology, Stockholm, Sweden), chosen for its flexibility in use across age groups and its robustness to children's head movement [3, 4]. Children are positioned in age-appropriate chairs. Five-month-olds and ten-month-olds are preferably seated in a maxi-cosi or infant car seat and three-years-olds on a children’s car seat. These chairs are mounted on a moveable platform to optimize the positioning between participant and eye tracker, and reduce child movement [5, 6]. Older children are seated on a regular desk chair and child movement is restrained with the use of a chin rest. During positioning, the relative position and orientation of participant and eye tracker are optimized to ensure stable tracking of the child's eyes. Hereafter, a 5-point operator-controlled calibration sequence is conducted (see Hessels et al., 2015b for details). Calibration stimuli (rotating, colorful spirals) are presented in the four corners and center of the screen in random order. The calibration output returned by the Tobii Pro SDK is examined for two features: Calibration points that are without data or with dispersed gaze data. These points are then re-calibrated by the operator until the calibration is deemed satisfactorily. Hereafter, or when the child starts to lose her attention, the experiment begins. Between experiments, re-positioning and re-calibration can be conducted if deemed necessary by the operator.
2. Computertasks: Some of the tasks conducted use a computer. In our center, until mid-2018, these computer tasks are executed on a Macbook Pro (model Early 2015 running Mac OS X 10.9 Mavericks) equipped with a 13" Retina display. A wired USB mouse is used for tasks requiring mouse operation. From mid-2018 onwards, the laptop has been replaced by a desktop (Dell Optiplex 7050, running Ubuntu 18.04 Bionic Beaver) with a 23" display. The mouse speed has been adjusted to match the set-up with the laptop as close as possible. The Peabody Picture Vocabulary task in ‘Around 3’ is administered using a touch screen (Dell S2240T 23.5"). Most tasks run in MATLAB (R2015a and R2018a), except for tasks from The University of Pennsylvania (i.e. the Computerized Neuropsychological Testing (PennCNP) task battery), which runs in a web browser (Firefox).
3. Video set-up: During the Parent- and Child Interaction (PCI) session, Hand Game, and delay of gratification tasks, the performances of the children and/or their parents are videotaped. These tasks take place in a room with curtain covered walls to optimize sound recording. The room is equipped with 3 Axis M5525-E PTZ network dome cameras with continuous 360⁰ pan, and a single fixed Axis M1054 network camera, that are connected to a desktop computer (Dell latitude E5540 or Dell Precision tower 5810) running Noldus Media recorder 3.0 or 4.0 software. Audio is recorded using a Lexcison Lambda Studio, Focusrite Scarlett 2i2, or Behringer U-phoria UMC202HD audio interface.
4. MRI and Mock scanner: Details about the MRI and Mock procedures have been described in detail in this issue (see also Buimer et al., this issue). Prior to participating in the MRI-session, children take part in an MRI-simulation procedure to allow them to acclimate to the scanner environment. The simulator is a replica of a standard scanner and mirrors all relevant aspects of the real scanner, such as speakers for simulating sounds and a table for simulating experiences the child has in the actual scanner. To mimic the real MRI session as closely as possible pre-recorded scanner sounds are played over a headphone, accompanied by slight trembling of the scanner. The child thus practices lying still and performing tasks while in the simulator. Only once the child is acclimatized to the scanner environment, so that both the child and parent are comfortable with the procedure, the child is taken to the actual scanner. If the child or parent is uncomfortable with any aspect of the procedure the MRI scan is cancelled. Prior to and right after the simulation scan both parents, researchers and children are asked to score the VAS scale to measure anxiety. Children are scanned on a Philips Ingenia CX 3 Tesla. The total MRI session lasts approximately 50 minutes. Once in the scanner, the child can see an LCD screen near the head through a 45-degree angle mirror fixed to the head coil (standard MRI equipment). Children are provided with earplugs and headphones, as the scanner can be fairly loud.

The technological effects to updates in scanner hardware and software are minimized by securing stable and dedicated machines and software. Quality control measures are generated automatically after each scanning session and results are accessible through an HTML-based portal on the local intranet for in-house viewing purposes. Every other week a proton spherical phantom is used to acquire a series of scans for control purposes to monitor changes over time as well as temporarily changes. However, over time scanner upgrades are unavoidable and required. To ensure acquisition stability we make sure these upgrades are done only after careful consideration of all parties concerned during two-weekly technical meeting, where MRI-acquisition of YOUth is discussed [see also in this issue:7].

**References:**

1. Albers, M., et al., *Intra- and interobserver agreement for fetal cerebral measurements in 3D-ultrasonography.* Hum Brain Mapp, 2018. **39**(8): p. 3277-3284.

2. van der Velde, B. and C. Junge, *Limiting Data Loss in Infant EEG: Putting hunches to the test.* Developmental Cognitive Neuroscience, 2020: p. 100809.

3. Hessels, R.S., et al., *Consequences of Eye Color, Positioning, and Head Movement for Eye-Tracking Data Quality in Infant Research.* Infancy, 2015. **20**(6): p. 601-633.

4. Niehorster, D.C., et al., *What to expect from your remote eye-tracker when participants are unrestrained.* Behav Res Methods, 2018. **50**(1): p. 213-227.

5. Hessels, R.S., et al., *Qualitative tests of remote eyetracker recovery and performance during head rotation.* Behav Res Methods, 2015. **47**(3): p. 848-59.

6. Hessels, R.S. and I.T. Hooge, *Eye tracking in developmental cognitive neuroscience–The good, the bad and the ugly.* Developmental cognitive neuroscience, 2019. **40**: p. 100710.

7. Buimer, E.E.L., et al., *Reliability of magnetic resonance imaging in YOUth.* Developmental Cognitive Neuroscience, 2020. **in submission**.
