# Supplementary material for: The YOUth study: Rationale, design, and study procedures
Source: Dev Cogn Neurosci. 2020 Oct 7;46:100868. doi: 10.1016/j.dcn.2020.100868 (PMC7575850; doi:10.1016/j.dcn.2020.100868)
Supplement: Supplementary file 2 [file mmc2.docx]

**Appendix 2: Extensive overview of questionnaires administered in YOUth.**

| **Questionnaire** | **Description** |
| --- | --- |
| **Rothbart’s Temperament Questionnaires [1-3]** | The Rothbart’s temperament questionnaires measure infant temperament in several domains. In YOUth Baby & Child the questionnaires (IBQ-R SF, ECBQ-SF, CBQ-SF, TMCQ) are age-dependent. To keep the burden for the primary caregiver as low as possible only the subscales Perceptual Sensitivity, Low Intensity Pleasure, Attentional Focusing, Inhibitory control, Impulsivity (only in Around 6) are assessed. In YOUth Child & Adolescent the full-scale EATQ-R-SF parent report form is used in all waves. To reduce the child’s burden in all waves of YOUth Child & Adolescent, only the YOUth main outcome variables (Inhibitory Control and Attention) of the children’s EATQ-R self-report are assessed. |
| **Ages & Stages Questionnaire: Social-Emotional Second Edition (ASQ-SE-2) [4, 5]** | The ASQ-SE-2 is a parent-report form that measures social and emotional development in young children. Depending on age the appropriate questionnaire is assessed. |
| **The Infant-Toddler Social & Emotional Assessment-Revised (ITSEA)** [6-8] | The ITSEA measures several dimensions of social-emotional problems and competencies in children aged 1 to 3 years. To reduce the burden of the primary caregiver, only the YOUth main outcome variables Empathy and Pro-social were assessed in Around 3. The ITSEA is replaced by Strengths and Difficulties Questionnaire (SDQ) as of September 2019, because the SDQ covers the entire range of our cohorts and can also reliably measure empathy and prosocial behavior. |
| **The Strengths and Difficulties Questionnaire (SDQ)** [9-11] | The SDQ is a brief behavioral screening questionnaire for children. In YOUth Baby & Child and YOUth Child & Adolescent primary caregivers complete the subscales Prosocial behavior and Peer problems, which are main outcome variables in YOUth. In addition, from Around 12 onwards a full scale child self-report measure is used. |
| **Interpersonal Reactivity Index (IRI) [12, 13]** | The IRI is a measure of empathy. In YOUth Baby & Child a parent report on child is used. In YOUth Child & Adolescent a child self-report measure is used. To reduce the participant’s burden only the subscales Perspective Taking and Empathic Concern are assessed. |
| **Demography questionnaire[14]** | The demography questionnaire is developed by Generation-R and provides information about the parental household, background, language, education, family relations, economic situation, religion. Each follow-up visit the parent is asked give updates whenever necessary. |
| **Work and Work environment questionnaire [14]** | The work and work environment questionnaire is developed by Generation-R and provides parental occupational information about, for example, type of work, work activities, working hours and occupational exposure to harmful substances or the reason for unemployment. |
| **Psychiatric family history questionnaire** | The psychiatric family history is a standardized questionnaire used by the Psychiatry department of the UMC Utrecht. The questionnaire provides information on psychiatric problems of first degree family members of the parents. In follow-up waves parents are asked to update the information whenever applicable. |
| **Adult self-report questionnaire (ASR) [15]** | The ASR measures aspects of adaptive functioning and problems in adults defined by the following scales: anxious/depressed, withdrawn, somatic complaints, thought problems, attention problems, aggressive behavior, rule-breaking behavior, and intrusive behavior. The questionnaire is completed by the mothers and fathers and is followed-up in most waves (see Table 5). |
| **Social Responsiveness Scale questionnaire (SRS-A)[16, 17]** | The SRS-A is a screening tool for autism spectrum disorders in adults focusing on social impairment. The questionnaire reliably measures autistic symptoms and characteristics in accordance with DSM-5 criteria. The SRS-A is a self-report measure for the parents. |
| **Strengths and Weaknesses of Attention-Deficit/Hyperactivity-symptoms and Normal-behaviors rating scale questionnaire (SWAN rating scale)[18-20]** | The SWAN is a rating scale for the primary caregiver to assess ADHD symptoms in children aged 6-17 years. |
| **Child Behavior Checklist questionnaire (CBCL)[21, 22]** | The CBCL is a standardized parent report form that measures skills and emotional and behavioral problems in youth. Two age dependent versions are used; 1.5-5 years and 6-18 years. |
| **Teacher’s Report Form (TRF) [23]** | The TRF is a standardized teacher report form that measures skills and emotional and behavioral problems in students. The questionnaire is completed by the teacher in Around 9. Due to a low response rate (<50%) this questionnaire was removed from the set in January 2019. |
| **Brief Symptom Inventory questionnaire (BSI)[24, 25]** | The BSI is a self-report measure to provide insight in psychopathological symptoms in adults and is completed by expecting parents. |
| **Edinburgh Postnatal Depression Scale questionnaire (EPDS)[26, 27]** | The EPDS measures risk factors for developing postpartum depression in women and is completed by the biological mother in Around 0. |
| **(Pre)pregnancy life style questionnaire** | The (pre)pregnancy life style questionnaire is developed by Generation R and contains variables about the intake of vitamins, coffee, tea, medication, exposure to harmful substances, alcohol, smoking and substance (ab)use prior to (both parents) or during pregnancy (only mothers) and is completed by the biological parents. |
| **General parental lifestyle questionnaire** | The general parental lifestyle questions about daily coffee and tea intake. The questions are the same as used in the (pre)pregnancy life style questionnaire. |
| **Smoking and substance (ab)use questionnaire** | The smoking and substance (ab)use questionnaire is a child self-report measure about smoking, alcohol intake, behavioral effects of alcohol (from Around 12 onwards), other substances (ab)use (from Around 12 onwards) and recalcitrant behavior (from Around 12 onwards). |
| **Parental smoking and substance (ab)use questionnaire** | The parental smoking and substance (ab)use questionnaire and contains questions about their current alcohol intake, smoking behavior and other substance (ab)use. The questions are the same as used in the (pre)pregnancy life style questionnaire. |
| **Nutrition questionnaire** | The nutrition questionnaire is developed by Wageningen University and is focused on eating pattern, breastfeeding and formula feeding, intake of energy, macronutrients, vitamin D, K (only in Around 0 4-6 months) and multivitamins. In YOUth Baby & Child the primary caregiver is asked to monitor the child’s eating behavior for one week. |
| **Food intake during pregnancy questionnaire[28]** | The Food Frequency questionnaire (FFQ) is focused on intake of energy, macronutrients, n-3 fatty acids, vitamin D, B-vitamins and folic acid during pregnancy. |
| **Physical Activity Questionnaire (PAQ) questionnaire[29-32]** | The PAQ is a self-report measure of physical activity in children. The questionnaire contains questions about free time physical activities, sports and intensity of physical activities. Based on age the PAQ-C (children aged 5-12) or PAQ-A (adolescents aged 12-17) is completed by children of YOUth Child & Adolescent. |
| **Short Questionnaire to Assess Health enhancing physical activity (SQUASH) questionnaire [33]** | SQUASH is a self-report measure of physical activity in terms of frequency, duration, intensity and type of activity. The SQUASH is completed by pregnant women, to measure physical activity during pregnancy. |
| **Sports and hobbies questionnaire** | The sports and hobbies questionnaire is created by GenerationR and measures the child’s sports and hobbies. The questionnaire is completed by the primary caregiver. |
| **Major Life Events questionnaire** | The Major Life Events questionnaire is a parent self-report that gives information about life events, e.g. divorce, financial problems, job promotion and health issues, that occurred over the past 12 months. |
| **Social Readjustment Rating Scale and List of long-term stressful conditions (Lijst met langdurig belastende omstandigheden) questionnaire[34]** | This questionnaire is a parent self-report composed by GenerationR and gives information on perceived stress, informal care and difficulties in the social environment. During pregnancy only mothers completed the questionnaire as an indicator of stress. From Around 6 onwards both parents are asked to complete the questionnaire as these stressors likely affect family functioning. |
| **Childhood memories questionnaire[35]** | The childhood memories questionnaire is a parent self-report that measures memories regarding their education. |
| **Childhood Trauma Questionnaire (CTQ)[36]** | The CTQ is a parental self-report measure that indicates traumatic life events during their youth, for instance physical abuse and neglect. |
| **Utrechtse Coping Lijst questionnaire (UCL)[37]** | The UCL is a parental self-report that measures their way of coping with problems and stressful situations. |
| **NEO-Five-Factor Inventory-3 questionnaire (NEO-FFI-3)[38]** | The NEO-FFI-3 is a self-report that measures the five domains of personality; Neuroticism, Extraversion, Openness, Agreeableness and Conscientiousness. The NEO-FFI-3 is completed by both parents in the first wave of both cohorts. |
| **Portrait Values Questionnaire - Revised (PVQ-RR)[39]** | The PVQ-RR is used to assess human values. Mothers and fathers in both cohorts are asked to complete the self-report. |
| **Self-Perception Profile for Adolescents (“Competentie Belevingsschaal”) questionnaire (CBSA))[40-42]** | The CBSA is a child self-report measure of competence in adolescents. In YOUth the revised edition is used from Around 9 onwards, as this version has better psychometric properties and is less time consuming than the original version[106]. |
| **Barrat Impulsiveness Scale -Brief questionnaire (BIS-Brief)[43-45]** | The Brief-BIS self-report is used to assess impulsiveness in the children from Around 9 onwards. |
| **Gender identity questionnaire (GI)** | The GI questionnaire contains two questions about how boy-like and how girl-like the child is according to the primary caretaker. |
| **Quick Big Five questionnaire (QBF)[46]** | The QBF is a short questionnaire to assess personality traits according to the five domains of personality; Neuroticism, Extraversion, Openness, Agreeableness and Conscientiousness. In YOUth Child & Adolescent the primary caretaker is asked to report on the child’s personality. |
| **Fiction questionnaire (FVL)[47]** | The Fiction questionnaire is used to measure the behavior regarding reading books, watching movies and series, playing computer games and using social media by the child. “How often do you read books?”, “What type of movies do you usually watch?” and “How often do you send a text, photo or movie with social media?” are some examples of the questions used in the questionnaire. In YOUth Baby & Child the primary caretaker reports on the child, in YOUth Child & Adolescent a child self-report is used. |
| **Media education questionnaire[48, 49]** | The Media education questionnaire is used to assess the way parents handle with the use of media (TV, games, Internet and social media) by the child. The questionnaire is completed by the primary caretaker. |
| **Network of relationships Social Provision Version - Short Form questionnaire (NRI-SPV-SF)[50-52]** | The NRI-SPV-SF measures features of close relationships. In YOUth Child & Adolescent a parent report on child is used to measure the support and negative interaction between the child and primary caretaker. A child self-report is used to measure the child’s relationship with friends in terms of support and negative interaction. |
| **Bullying questionnaire** | The bullying questionnaire is a parent report on bullying behavior of and towards the child. An example question is “How often in the past months is your son/daughter bullied by spitting, beating, kicking or pinching?”. |
| **Social Support List questionnaire (SSL)[53, 54]** | The SSL measures social support in terms of positive and negative interaction with other people and the resemblance with one’s personal needs. Social support interacts with the way people deal with stressful situations, e.g. the birth of a child. In YOUth the SSL is therefore primarily used together with the EPDS as a confounding risk factor for postpartum depression in the biological mother. |
| **Sleep Self Report questionnaire (SSR)[55]** | The SSR is a self-report measure for children and is used to measure overall sleep problems in children in Around 9. |
| **Promis Sleep Item Bank questionnaire[56, 57]** | Three scales of the PROMIS Pediatric Item Bank are administered in YOUth, namely the Sleep Disturbance scale (e.g. difficulties falling asleep), Sleep Practices (e.g. bedtime routine), and Sleep related impairment (e.g. daytime sleepiness). The scales are self-report measures and used from Around 12 onwards in children. |
| **Pittsburgh Sleep Quality Index questionnaire (PSQI)[58]** | The PSQI is a self-report measure that indicates sleep quality defined by perceived sleep quality, sleep latency, duration, efficiency, disturbances, use of medication and daytime dysfunctioning. The questionnaire is completed by the mother during pregnancy. |
| **Children's Sleep Habits Questionnaire (CSHQ)[55, 59]** | The CSHQ is a parent report measure to examine sleep habits and difficulties with sleep in children. |
| **Parental Control Scale questionnaire (PCS)[60-62]** | The PCS is a child self-report measure that indicates the perceived parental control. |
| **Child Report of Parenting Behavior Inventory questionnaire (CRPBI)[63]** | The CRPBI measures the child’s perception of parental attitude and behavior with three subscales, namely Acceptance/involvement, Psychological autonomy, and Strictness/supervision. To reduce the burden of the children, only the Strictness/supervision scale is used in YOUth. |
| **Alabama Parenting Questionnaire (APQ)[64-68]** | The APQ is parent report on child (6-18 years) and measures five domains of parenting, namely Positive involvement with children, Supervision and monitoring, Use of positive discipline techniques, Consistency in the use of such discipline, and Use of corporal punishment. In YOUth only the Use of corporal punishment is assessed by the primary caretaker. |
| **Child‐Rearing Questionnaire (NOV)[69]** | The NOV is a self-report measure to assess parenting behavior by means of Responsiveness, Autonomy, Consistency and Monitoring. In YOUth Responsiveness is completed by the primary caretaker. |
| **Parenting Dimensions Inventory questionnaire (PDI)[70]** | The PDI is a self-report measure of parenting style, in YOUth the domain Consistent disciplining is measured in the primary caretaker. |
| **Parenting stress index (Nijmeegse Ouderlijke Stress Index, NOSI)[71, 72]** | The NOSI is a self-report measure, that measures the perceived level of stress in the parent-child relationship. In YOUth the subscales Acceptance (of the child and expectation of the parent regarding physical, intellectual and emotional characteristics) and Competence (the sense of having enough skills and ways to cope with the child) are assessed by the primary caretaker. |
| **Parental Monitoring Questionnaire (Vragenlijst Toezicht houden(VTH)) [73]** | The VTH is a six-item parental self-report measure to assess parental behavioral control. |
| **Parenting Practices questionnaire (PP):Brown, 1993; Kerr, 2000; Stattin, 2000; Keijsers, 2009}** | The PP is a child self-report measure about the relation with their parent in terms of the child’s disclosure. |
| **Comprehensive Early Childhood Parenting Questionnaire (CECPAQ)[74]** | The CECPAQ is a parent report measure of parenting behavior towards children aged 1-4 years. The questionnaire has five domains, namely support, stimulation, structure, harsh discipline and positive discipline. The CECPAQ is completed by the primary caretaker. |
| **Pubertal development scale questionnaire (PDS)[75]** | The PDS is a child self-report measure of pubertal maturation and is administered from Around 9 onwards. |
| **Sexual development questionnaire[76-79]** | The sexual development questionnaire is a child self-report measure that contains questions about love, relationships and (online) sexual behavior. |
| **Language situation questionnaire** | The Language situation questionnaire is a parent report on the spoken languages in the child’s environment, e.g. at home or at the daycare. |
| **Clinical Evaluation of Language Fundamentals questionnaire (CELF){wiig, 2004; Wiig, 2012; Kort, 2008** | The CELF is a parent report measure to test language abilities in children (3-16 years). In YOUth the Pragmatics list of the CELF Preschool-2-NL and the CELF-4-NL is used. This list measures non-verbal communication skills, the ability to ask for, react to and provide information, and communicative skills and routines. |
| **Communicative Development Inventory questionnaire (N-CDI)[80]** | The N-CDI is a parent report that measures the development of communicative skills in children (8-30 months). In YOUth the N-CDI-1 is used in Around 0 (9-11 months) and a combination of the N-CDI-2 and N-CDI-3 is used in Around 3. |
| **Daycare questionnaire** | The daycare questionnaire provides information about time spend with other caretakers (e.g. nanny) and whether other children are present during this time. |
| **Periconceptual health questionnaire** | The periconceptual health questionnaire provides information about the pregnancy, like due date, fertility treatment, miscarriage or still birth, prior obstetric outcomes and complications in prior pregnancies. |
| **Obstetric outcome questionnaire** | The obstetric outcome questionnaire contains questions about labor (e.g. duration) and birth (e.g. Apgar score) of the child that participates in YOUth and is completed by the mother in YOUth Baby & Child and the primary caretaker in YOUth Child & Adolescent. |
| **General health questionnaire** | The General health questionnaire is a parent report on the child’s mental (based on DSM-V classification) and physical health (e.g. epilepsy and asthma). |
| **General parental health questionnaire** | The General parental health questionnaire contains information about birth (e.g. weight), sexual contacts, sexually transmitted diseases and other medical information (e.g. high blood pressure, epilepsy and auto-immune diseases) and is completed by both parents. |
| **Medical family history questionnaire** | The Medical family history questionnaire provides information on medical conditions (e.g. cancer, migraine and cardiovascular diseases) in first degree family members of the parents. |
| **Anthropometry and vaccinations questionnaire** | In this questionnaire the primary caretaker of YOUth Baby & Child is asked to provide information on the child’s growth, weight gain measured at an infant welfare center, and participation in the Dutch National Immunization Programmed (Rijksvaccinatieprogramma). Each visit the primary caretaker is asked to update the information. |

**References**

1. Putnam, S.P., et al., *Development and assessment of short and very short forms of the infant behavior questionnaire-revised.* J Pers Assess, 2014. **96**(4): p. 445-58.

2. Putnam, S.P. and M.K. Rothbart, *Development of short and very short forms of the Children's Behavior Questionnaire.* J Pers Assess, 2006. **87**(1): p. 102-12.

3. Ellis, L.K. and M.K. Rothbart. *Revision of the Early Adolescent Temperament Questionnaire. Poster presentation*. in *The biennial meeting of the Society for Research in Child Development*. 2001. Minneapolis, USA.

4. Squires, J., D. Bricker, and E. Twombly, *Ages & Stages Questionnaires-Social-Emotional: A parent-completed, childmonitoring system for social-emotional behaviors*. 2002, Baltimore: Paul H. Brookes Publishing Co., Inc.

5. Steenis, L.J., et al., *Parental and professional assessment of early child development: the ASQ-3 and the Bayley-III-NL.* Early Hum Dev, 2015. **91**(3): p. 217-25.

6. Visser, J.C., et al., *Assessment of psychopathology in 2- to 5-year-olds: Applying the Infant-Toddler Social Emotional Assessment.* Infant Ment Health J, 2010. **31**(6): p. 611-629.

7. Carter, A.S., et al., *The Infant-Toddler Social and Emotional Assessment (ITSEA): factor structure, reliability, and validity.* J Abnorm Child Psychol, 2003. **31**(5): p. 495-514.

8. Briggs-Gowan, M.J. and A.S. Carter, *Preliminary acceptability and psychometrics of the Infant-Toddler Social and Emotional Assessment (ITSEA): A new adult-report questionnaire.* Infant Ment Health J, 1998. **19**(4): p. 422-445.

9. van Widenfelt, B.M., et al., *Dutch version of the Strengths and Difficulties Questionnaire (SDQ).* Eur Child Adolesc Psychiatry, 2003. **12**(6): p. 281-9.

10. Goodman, R., *The Strengths and Difficulties Questionnaire: a research note.* J Child Psychol Psychiatry, 1997. **38**(5): p. 581-6.

11. Goodman, R., H. Meltzer, and V. Bailey, *The Strengths and Difficulties Questionnaire: A pilot study on the validity of the self-report version.* Eur Child Adolesc Psychiatry, 1998. **7**(3): p. 125-130.

12. De Corte, K., et al., *Measuring Empathic Tendencies: Reliability and Validity of the Dutch Version of the Interpersonal Reactivity Index.* Psychologica Belgica, 2007. **47**(4): p. 235-260.

13. Davis, M., *A Multidimensional Approach to Individual Differences in Empathy.* JSAS Catalog Sel. Doc. Psychol., 1980. **10**.

14. Jaddoe, V.W., et al., *The Generation R Study: Design and cohort profile.* Eur J Epidemiol, 2006. **21**(6): p. 475-84.

15. Achenbach, T.M. and L.A. Rescorla, *Manual for the ASEBA Adult Forms & Profiles.*, R.C.f.C. University of Vermont, Youth, and Families., Editor. 2003: Burlington, VT.

16. Constantino, J.N. and C.P. Gruber, *Social Responsiveness Scale: Manual.*, W.P. Services, Editor. 2005: Los Angeles, CA.

17. Noens, I., W. De la Marche, and E. Scholte, *SRS-A Screeningslijst voor autismespectrumstoornissen. Handleiding.* . 2012: Amsterdam.

18. Polderman, T.J.C., et al., *Across the continuum of attention skills: a twin study of the SWAN ADHD rating scale.* Journal of Child Psychology and Psychiatry, 2007. **48**(11): p. 1080-1087.

19. Arnett, A.B., et al., *The SWAN captures variance at the negative and positive ends of the ADHD symptom dimension.* J Atten Disord, 2013. **17**(2): p. 152-62.

20. Lakes, K.D., J.M. Swanson, and M. Riggs, *The reliability and validity of the English and Spanish Strengths and Weaknesses of ADHD and Normal behavior rating scales in a preschool sample: continuum measures of hyperactivity and inattention.* J Atten Disord, 2012. **16**(6): p. 510-6.

21. Verhulst, F.C., J. Van Der Ende, and H.M. Koot, *Handleiding voor de CBCL/1.5-5 (Manual for the CBCL/1.5-5)* 1996, Erasmus University/Department of Child and Adolescent Psychiatry, Sophia Children’s Hospital/AZR/EUR: Rotterdam.

22. Verhulst, F.C., J. Van Der Ende, and H.M. Koot, *Handleiding voor de CBCL/4-18 (Manual for the CBCL/4-18)*. 1996, Erasmus University/Department of Child and Adolescent Psychiatry, Sophia Children’s Hospital/AZR/EUR: Rotterdam.

23. Achenbach, T.M., *Manual for the Teacher's Report Form and 1991 profile*. 1991: Univ Vermont/Department Psychiatry.

24. De Beurs, E.J., *Brief Symptom Inventory manual*, t.N. Dutch translation PITS BV Leiden, Editor. 2006.

25. Derogatis, L.R. and N. Melisaratos, *The brief symptom inventory: an introductory report.* J Psychological medicine, 1983. **13**(3): p. 595-605.

26. Pop, V.J., I.H. Komproe, and M.J. Vanson, *Characteristics of the Edinburgh Post Natal Depression Scale in the Netherlands.* Journal of Affective Disorders, 1992. **26**(2): p. 105-110.

27. Cox, J.L., J.M. Holden, and R. Sagovsky, *Detection of Postnatal Depression - Development of the 10-Item Edinburgh Postnatal Depression Scale.* British Journal of Psychiatry, 1987. **150**: p. 782-786.

28. Health, N.I.f.P., et al., *National dietary survey in 2012‐2016 on the general population aged 1‐79 years in the Netherlands.* J EFSA Supporting Publications, 2018. **15**(9): p. 1488E.

29. Bervoets, L., et al., *Reliability and validity of the Dutch physical activity questionnaires for children (PAQ-C) and adolescents (PAQ-A).* J Archives of Public Health, 2014. **72**(1): p. 47.

30. Kowalski, K.C., P.R. Crocker, and R.M. Donen, *The physical activity questionnaire for older children (PAQ-C) and adolescents (PAQ-A) manual.* J College of Kinesiology, University of Saskatchewan, 2004. **87**(1): p. 1-38.

31. Kowalski, K.C., P.R. Crocker, and R.A. Faulkner, *Validation of the physical activity questionnaire for older children.* J Pediatric exercise science, 1997. **9**(2): p. 174-186.

32. Kowalski, K.C., P.R. Crocker, and N.P. Kowalski, *Convergent validity of the physical activity questionnaire for adolescents.* J Pediatric exercise science, 1997. **9**(4): p. 342-352.

33. Wendel-Vos, G.W., et al., *Reproducibility and relative validity of the short questionnaire to assess health-enhancing physical activity.* J Journal of clinical epidemiology, 2003. **56**(12): p. 1163-1169.

34. Hendriks, A., J. Ormel, and G. Van de Willige, *Long lasting difficulties measured with a self-assessment questionnaire and semi structured interview: a theoretical and empirical comparison.* Gedrag en Gezondheid, 1990. **18**: p. 273-283.

35. Arrindell, W.A., et al., *Cross-national invariance of dimensions of parental rearing behaviour: comparison of psychometric data of Swedish depressives and healthy subjects with Dutch target ratings on the EMBU.* The British Journal of Psychiatry, 1986. **148**(3): p. 305-309.

36. Bernstein, D.P., et al., *Initial reliability and validity of a new retrospective measure of child abuse and neglect.* The American journal of psychiatry, 1994. **151**(8): p. 1132-1136.

37. Schreurs, P.J.G., et al., *De Utrechtse coping lijst: omgaan met problemen en gebeurtenissen*. 1993, Amsterdam, the Netherlands: Pearson Assessment & Information B.V.

38. Costa, P.T. and R.R. MacCrae, *Revised NEO personality inventory (NEO PI-R) and NEO five-factor inventory (NEO-FFI): Professional manual*. 1992: Psychological Assessment Resources, Incorporated.

39. Schwartz, S.H., et al., *Refining the theory of basic individual values.* Journal of personality and social psychology, 2012. **103**(4): p. 663.

40. Treffers, P., et al., *Competentie belevingsschaal voor Adolescenten.* Tijdschrift voor Psychiatrie, 2004. **7**: p. 468-469.

41. Wichstraum, L., *Harter's Self-Perception Profile for Adolescents: Reliability, validity, and evaluation of the question format.* J Pers Assess, 1995. **65**(1): p. 100-116.

42. Wiegerink, D.J., et al., *Social, intimate and sexual relationships of adolescents with cerebral palsy compared with able-bodied age-mates.* Journal of rehabilitation medicine, 2008. **40**(2): p. 112-118.

43. Patton, J.H., M.S. Stanford, and E.S. Barratt, *Factor structure of the Barratt impulsiveness scale.* Journal of clinical psychology, 1995. **51**(6): p. 768-774.

44. Stanford, M.S., et al., *Fifty years of the Barratt Impulsiveness Scale: An update and review.* Personality and individual differences, 2009. **47**(5): p. 385-395.

45. Steinberg, L., et al., *New tricks for an old measure: The development of the Barratt Impulsiveness Scale–Brief (BIS-Brief).* Psychological assessment, 2013. **25**(1): p. 216.

46. Vermulst, A. and J. Gerris, *Quick Big Five persoonlijkheidstest handleiding (Quick Big Five personality-test manual).* 2009, 's-Hertogenbosch: Malmberg.

47. Fikkers, K., et al., *Double dose: High family conflict enhances the effect of media violence exposure on adolescents’ aggression.* Societies, 2013. **3**(3): p. 280-292.

48. Valkenburg, P.M., et al., *Developing and validating the perceived parental media mediation scale: A self-determination perspective.* Human Communication Research, 2013. **39**(4): p. 445-469.

49. Valkenburg, P.M., et al., *Developing a scale to assess three styles of television mediation:“Instructive mediation,”“restrictive mediation,” and “social coviewing”.* Journal of broadcasting & electronic media, 1999. **43**(1): p. 52-66.

50. Furman, W. and D. Buhrmester, *Children's perceptions of the personal relationships in their social networks.* Dev Psychol, 1985. **21**(6): p. 1016.

51. De Goede, I.H., et al., *Linkages over time between adolescents' relationships with parents and friends.* Journal of youth and adolescence, 2009. **38**(10): p. 1304-1315.

52. Furman, W. and D. Buhrmester, *Age and sex differences in perceptions of networks of personal relationships.* Child Dev, 1992. **63**(1): p. 103-115.

53. Bridges, K.R., R. Sanderman, and E. Van Sonderen, *An English language version of the social support list: preliminary reliability.* Psychological Reports, 2002. **90**(3): p. 1055-1058.

54. van Sonderen, E., *Sociale Steun Lijst–Interacties (SSL-I) en Sociale Steun Lijst-Discrepanties (SSL-D)*. 1997, Groningen: Centrum voor Gezondheidsvraagstukken Rijksuniversiteit Groningen.

55. van Litsenburg, R.R.L., et al., *Sleep habits and sleep disturbances in Dutch children: a population-based study.* European journal of pediatrics, 2010. **169**(8): p. 1009-1015.

56. Haverman, L., et al., *Dutch–Flemish translation of nine pediatric item banks from the Patient-Reported Outcomes Measurement Information System (PROMIS)®.* Quality of Life Research, 2016. **25**(3): p. 761-765.

57. van Kooten, J.A., et al., *Validation of the PROMIS Sleep Disturbance and Sleep-Related Impairment item banks in Dutch adolescents.* Quality of Life Research, 2018. **27**(7): p. 1911-1920.

58. Buysse, D.J., et al., *The Pittsburgh Sleep Quality Index: a new instrument for psychiatric practice and research.* Psychiatry Res, 1989. **28**(2): p. 193-213.

59. Owens, J.A., A. Spirito, and M. McGuinn, *The Children's Sleep Habits Questionnaire (CSHQ): psychometric properties of a survey instrument for school-aged children.* Sleep-New York-, 2000. **23**(8): p. 1043-1052.

60. Barber, B.K., *Intrusive parenting: How psychological control affects children and adolescents*. 2002, Washington: American Psychological Association.

61. Barber, B.K., *Parental psychological control: Revisiting a neglected construct.* Child Dev, 1996. **67**(6): p. 3296-3319.

62. Barber, B.K., J.E. Olsen, and S.C. Shagle, *Associations between parental psychological and behavioral control and youth internalized and externalized behaviors.* Child Dev, 1994. **65**(4): p. 1120-1136.

63. Schaefer, E., *(1965a). Children" s reports of parental behavior: An inventory. Child Development, 36, 413-424.* 1965.

64. Shelton, K.K., P.J. Frick, and J. Wootton, *Assessment of parenting practices in families of elementary school-age children.* Journal of clinical child psychology, 1996. **25**(3): p. 317-329.

65. Frick, P.J., R.E. Christian, and J.M. Wootton, *Age trends in the association between parenting practices and conduct problems.* Behavior modification, 1999. **23**(1): p. 106-128.

66. Fox, R., *Parent behaviour checklist. Brandon, Vermont*. 1994, Clinical Psychology Publishing Co. Inc.

67. Verhoeven, M., et al., *Parenting during toddlerhood: Contributions of parental, contextual, and child characteristics.* Journal of Family Issues, 2007. **28**(12): p. 1663-1691.

68. Essau, C.A., S. Sasagawa, and P.J. Frick, *Psychometric properties of the Alabama parenting questionnaire.* Journal of Child and Family Studies, 2006. **15**(5): p. 595-614.

69. Gerris, J., et al., *Parenting in Dutch families: A representative description of validated concepts representing characteristics of parents, children, the family as a system and socio-cultural value orientations*. 1993, Nijmegen, The Netherlands: University of Nijmegen, Institute of Family Studies.

70. Slater, M.A. and T.G. Power, *Multidimensional assessment of parenting in single-parent families.* Advances in family intervention, assessment, and theory, 1987. **4**: p. 197-228.

71. De Brock, A., et al., *NOSI: Nijmeegse ouderlijke stress index*. 1992, Lisse: Swets en Zeitlinger.

72. Abidin, R.R., *Parenting Stress Index--Manual (PSI)*. 1983, Charlottesville: CPPC Tests.

73. Dekovic, M., *Vragenlijst Toezicht Houden (VTH) (Parental Monitoring (Questionnaire)*. 1996, Utrecht: Utrecht University.

74. Verhoeven, M., et al., *Development and initial validation of the comprehensive early childhood parenting questionnaire (CECPAQ) for parents of 1-4 year-olds.* European Journal of Developmental Psychology, 2017. **14**(2): p. 233-247.

75. Carskadon, M.A. and C. Acebo, *A Self-Administered Rating-Scale for Pubertal Development.* Journal of Adolescent Health, 1993. **14**(3): p. 190-195.

76. Van de Bongardt, D., E. Reitz, and M. Deković, *Vroeg seksueel debuut in Nederland: een longitudinale studie naar de rol van individuele kenmerken, opvoeding en leeftijdgenoten.* Kind & Adolescent, 2013. **33**: p. 194-205.

77. Baams, L., et al., *De rol van persoonlijkheid in de ontwikkeling van seksueel gedrag van adolescenten.* Kind & Adolescent, 2012. **33**(4): p. 206-217.

78. Doornwaard, S.M., et al., *Ontwikkelingstrajecten in en voorspellers voor het gebruik van seksueel expliciet internetmateriaal.* Kind & Adolescent, 2012. **33**(4): p. 226-238.

79. de Graaf, H. and I. Vanwesenbeeck, *'Seks is een game’. Gewenst en ongewenst seksueel gedrag van jongeren op het internet*. 2006, Utrecht: Rutgers Nisso Groep.

80. Zink, I. and M. Lejaegere, *N-CDI’s: korte vormen. Aanpassing en hernormering van de MacArthur Short Form Vocabulary Checklist van Fenson et al.* 2003, Leuven/Leusden: Acco.
